# Cell jamming transition is regulated by mitochondrial pyruvate transport and endocytosis

**DOI:** 10.1073/pnas.2617216123

**Published:** 2026-10-07

**Authors:** Alexandra Bermudez, Zoe Latham, Johnny Diaz, Weihong Yan, Jerry Chen, Dapeng Bi, Andrew S. Goldstein, Jimmy K. Hu, Neil Y. C. Lin

**Affiliations:** ^a^ Mechanical and Aerospace Engineering Department, University of California, Los Angeles, CA 90095; ^b^ Bioengineering Department, University of California, Los Angeles, CA 90095; ^c^ Department of Molecular, Cell, and Developmental Biology, University of California, Los Angeles, CA 90095; ^d^ Department of Chemistry and Biochemistry, University of California, Los Angeles, CA 90095; ^e^Department of Physics, Northeastern University, Boston, MA 02115; ^f^ Division of Biosystems and Function, School of Dentistry, University of California, Los Angeles, CA 90095; ^g^ Broad Stem Cell Center, University of California, Los Angeles, CA 90095

**Keywords:** epithelial jamming transition, collective cell migration, mechanobiology

## Abstract

Epithelial tissues undergo dynamic transitions between fluid-like collective motion and mechanically jammed states during development, injury repair, and disease progression. However, the cellular programs that drive these transitions and regulate collective behavior remain unclear. Using a controlled crowding model integrated with live-cell imaging and time-resolved multiomics, we demonstrate that epithelial crowding triggers early metabolic changes characterized by increased mitochondrial pyruvate anaplerosis that precedes the jamming transition. Restricting mitochondrial pyruvate import increased collective cell motility and delayed jamming in crowded monolayers. This unjammed state is driven by enhanced cytoskeletal remodeling and requires RhoA-myosin II activity. Mechanistically, we show that elevated cytoskeletal signaling promotes macropinocytic uptake, which serves as a required feedback loop to maintain motility. These findings identify mitochondrial pyruvate utilization as an important regulatory input linking metabolic remodeling to the endocytic control of epithelial fluidity.

## Significance

Epithelial tissues transition between fluid-like motility and solid-like stability during development and repair. While the physical mechanics governing this jamming transition are established, the cell-intrinsic biological pathways controlling it remain largely undefined. This study identifies mitochondrial pyruvate anaplerosis as an important regulatory input that suppresses collective epithelial motility during tissue crowding. Restricting mitochondrial pyruvate import delays jamming, sustaining a fluidized monolayer state through enhanced RhoA-actomyosin activity and a necessary compensatory increase in macropinocytosis. These results establish a mechanistic link between intracellular metabolic routing and the endocytic control of tissue-scale mechanics. This work provides a biological framework for understanding how epithelia integrate metabolic cues to autonomously modulate their macroscopic physical state.

Epithelial tissues possess the remarkable ability to transition between fluid-like, motile collectives and solid-like, mechanically stable sheets. This ability enables the dynamic morphological and structural transformations essential for organ morphogenesis, injury repair, and tissue homeostasis (1–5). As cells multiply within confined environments, increasing density drives extensive cytoskeletal remodeling and junction maturation. These changes trigger the contact inhibition of both locomotion and proliferation (6, 7), which subsequently reduces cell motility and rearrangements to solidify the epithelium and preserve tissue architecture (8–10). This jamming transition is critical for stabilizing tissues during key developmental events, such as the conclusion of *Drosophila* wing elongation (11), tissue convergent extension (12), and the final phases of wound healing (13). However, despite the well-characterized physical principles governing these states, the cell-intrinsic regulatory mechanisms and biological pathways that drive the jamming–unjamming transition remain a fundamental open question.

By integrating principles from mechanobiology and soft-matter physics, recent research has established a robust biophysical framework to characterize collective cell dynamics during jamming–unjamming transitions. This framework highlights the role of tissue-scale mechanics, specifically how cell packing, force transmission, and geometric deformation govern transition states (14–17). Beyond these intrinsic mechanics, collective migration is modulated by a broad spectrum of extrinsic regulators, ranging from physical inputs such as substrate properties (18–21) and compressive stress (22) to environmental stimuli including morphogen gradients (23), electrotaxis (24), and interstitial flow (25, 26). Crucially, in many contexts, collective migration is facilitated not by an epithelial–mesenchymal transition (EMT), but by a physical unjamming transition (UJT) (22, 27–30). This UJT allows tissues to adopt a fluid, motile state while strictly maintaining their epithelial molecular identity and barrier integrity.

While the physical principles of epithelial jamming are well characterized, the cell-intrinsic pathways that transduce crowding cues into behavioral shifts remain incompletely defined (4, 10). Cadherin-mediated adhesion is a known rheological rheostat for the fluid-to-solid transition (30); however, because upstream signals dictate adhesion dynamics (31), the primary sensing mechanisms governing unjamming remain elusive. Elucidating these pathways is essential, as morphogenesis, wound healing, and malignancy all depend on mechanical state changes regulated by diverse cellular programs, including metabolism (32, 33). For instance, the fluidized state in wound repair requires elevated glycolytic activity (34), whereas metabolic reprogramming during cancer invasion scales with the mechanical properties of the tumor microenvironment (35–38). Despite these correlations, the precise mechanisms by which intracellular metabolism integrates crowding-induced mechanical information to drive the jamming transition remain an unresolved frontier.

Here, we address this question using a controlled epithelial crowding model based on Madin–Darby canine kidney (MDCK) monolayers that progress from sparse, motile collectives to densely packed, jammed sheets. By integrating live-cell imaging with time-resolved transcriptomics and metabolomics, we captured the evolving relationship between cell behavior, transcriptional remodeling, and metabolic state during the jamming transition. This multimodal framework allows us to connect molecular regulation to emergent collective dynamics and helps bridge a long-standing disconnect between physical and biological views of epithelial jamming. Using this cross-discipline approach, we identify pyruvate anaplerosis as a central response to crowding that modulates actomyosin activity and suppresses endocytosis, thereby promoting the cessation of cell movement and the establishment of a jammed state. This intrinsic program coordinates metabolic and cytoskeletal responses to increasing cell density and provides a foundation for understanding how epithelial tissues autonomously regulate transitions between fluid and solid states.

## Results

### Early Metabolic Remodeling Precedes Adhesion and YAP Translocation During Cell Crowding.

**Fig. 1.**
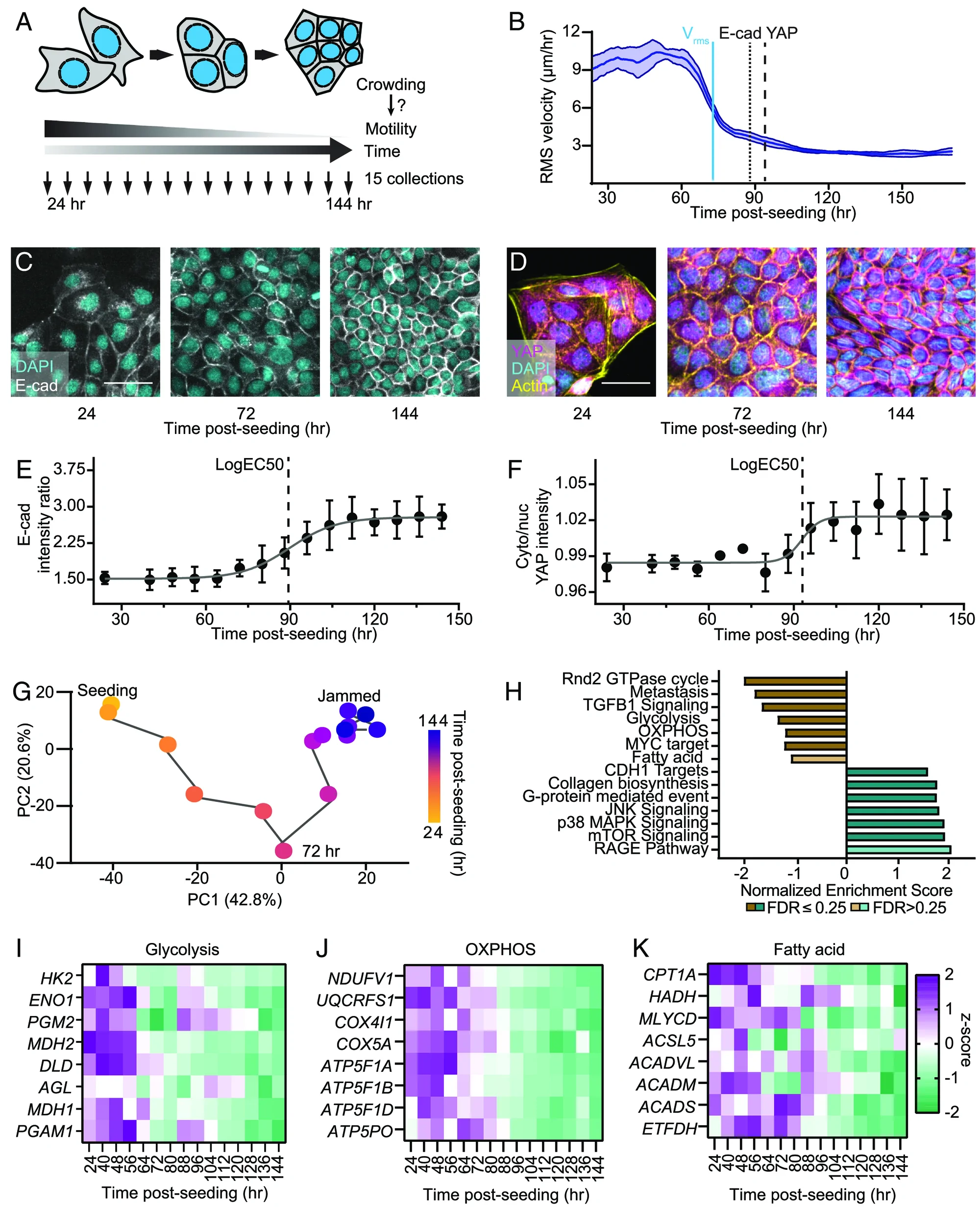
Metabolic remodeling precedes adhesion and YAP transitions in crowding Epithelia. (*A*) Schematic of the experimental design using the MDCK cell jamming model. Subconfluent cells progressively undergo crowding, during which cell velocity, E-cadherin and YAP levels, and RNA expression were quantified across 15 time points. (*B*) RMS cell velocity ($v_{\mathrm{rms}}$) measured from the same time series exhibits an earlier decline (≈70 h) than the E-cadherin and YAP transitions. (*C*) E-cadherin immunofluorescence showing the progressive maturation of adherens junctions during crowding. (Scale bar, 50 μm.) (*D*) YAP immunofluorescence demonstrating the gradual enrichment of YAP in the cytoplasm. (Scale bar, 50 μm.) (*E*) Quantification of the junction-to-cytoplasm E-cadherin intensity ratio reveals a time-dependent increase following a sigmoidal trend (LogEC_50_≈90 h). n = 40 where n = cells analyzed. (*F*) Quantification of the cytoplasmic-to-nuclear YAP intensity ratio shows a sigmoidal increase (LogEC_50_≈ 90 h), indicating reduced nuclear YAP localization. n = 44, 43, 42, 44, 44, 41, 72, 83, 48, 55, 42, 46, 100, 91, and 47, for each time point, respectively, where n = cells analyzed. (*G*) Principal component analysis of bulk RNA-seq profiles depicts a nonlinear transcriptional trajectory with an inflection near 70 h. (*H*) Gene set enrichment analysis between T1 and T5 identifies pathways associated with epithelial differentiation, signaling, and metabolism that significantly change over time. (*I*–*K*) Expression dynamics of key metabolic gene sets, glycolysis, OXPHOS, and fatty-acid metabolism, show coordinated downregulation beginning near 70 h, preceding adherens junction maturation and YAP relocalization. N = 3 independent biological replicates were used for all analyses, except for *B* (N = 6) and *E*, with each replicate representing a separate well. For *E*, n = 40 cells were analyzed from one well per time point; this analysis is descriptive.

Cell crowding is a dynamic process in which coordinated changes in local cell density, junction organization, and molecular signaling progressively remodel tissue mechanics. While understanding how these changes evolve is essential for elucidating the fluid-to-solid jamming transition, a detailed temporal description of these events remains lacking. To capture this evolution, we employed an MDCK epithelial monolayer model, tracking cells from a sparse, subconfluent state to a densely packed, jammed configuration. Across fifteen evenly spaced time points (T1–T15), we examined three key processes linked to jamming: cell movement, E-cadherin–based adhesion, and YAP localization (Fig. 1*A*). We quantified the RMS cell velocity ($V_{\mathrm{rms}}$) via particle image velocimetry, observing a characteristic decay and plateau that confirmed our time series captured the full jamming process (Fig. 1*B* and Movie S1). Simultaneously, we monitored the maturation of E-cadherin junctions through the immunofluorescence junctional contrast normalized to the cytoplasmic background (Fig. 1*C* and *SI Appendix*, Fig. S1*A*) and tracked the mechanoresponsive inactivation of YAP via its cytoplasmic-to-nuclear intensity ratio (Fig. 1*D* and *SI Appendix*, Fig. S1*B*).

Notably, we found that both the initiation of E-cadherin junction maturation (Fig. 1*E*) and the decrease in nuclear YAP (Fig. 1*F*) occurred at approximately 90 h postseeding, whereas cell motility ($V_{\mathrm{rms}}$) began to decline roughly 30 h before these events (Fig. 1*B*). This temporal sequence, where motility loss precedes junctional maturation and YAP relocalization, is consistent with previous studies (39, 40) showing that epithelial cells lose motility at intermediate densities well before reaching high-density confluent states. Collectively, these findings suggest that the initial regulation of collective cell motility during the jamming transition involves mechanisms independent of adherens junction maturation and YAP-dependent transcriptional control.

To identify the molecular mechanisms governing this early decline in collective motility, we performed time-series bulk RNA sequencing (RNA-seq) across the fifteen time points previously described (T1–T15), with three biological replicates per point. Principal component analysis (PCA) revealed a nonlinear transcriptional trajectory with a distinct inflection point along the second principal component (PC2) at approximately 70 h. This shift coincided with the onset of E-cadherin junction maturation and the reduction in nuclear YAP localization (Fig. 1*G*). Crucially, we observed pronounced and sustained transcriptional changes during the early crowding process (T1–T5) that preceded this 70 h inflection point (Fig. 1*H*).

Gene set enrichment analysis identified significant remodeling of cellular metabolism, specifically within glycolysis (Fig. 1*I* and *SI Appendix*, Fig. S1*C*), oxidative phosphorylation (OXPHOS; Fig. 1*J* and *SI Appendix*, Fig. S1*D*), and fatty acid metabolism (Fig. 1*K* and *SI Appendix*, Fig. S1*E*). Heatmap visualizations of z-scored metabolic genes revealed a consistent downregulation during this early phase of crowding, occurring well before the major E-cadherin and YAP transitions (Fig. 1*I*–*K*). Similar changes were observed in other gene sets, including those associated with TGF-β1 signaling and E-cadherin loss (*SI Appendix*, Fig. S1 *F* and *G*). Together, these patterns suggest that metabolic remodeling is not merely a consequence of jamming, but rather an early regulatory event in the crowding-induced transition.

### Pyruvate Anaplerosis Limits Collective Cell Motility During Epithelial Crowding.

**Fig. 2.**
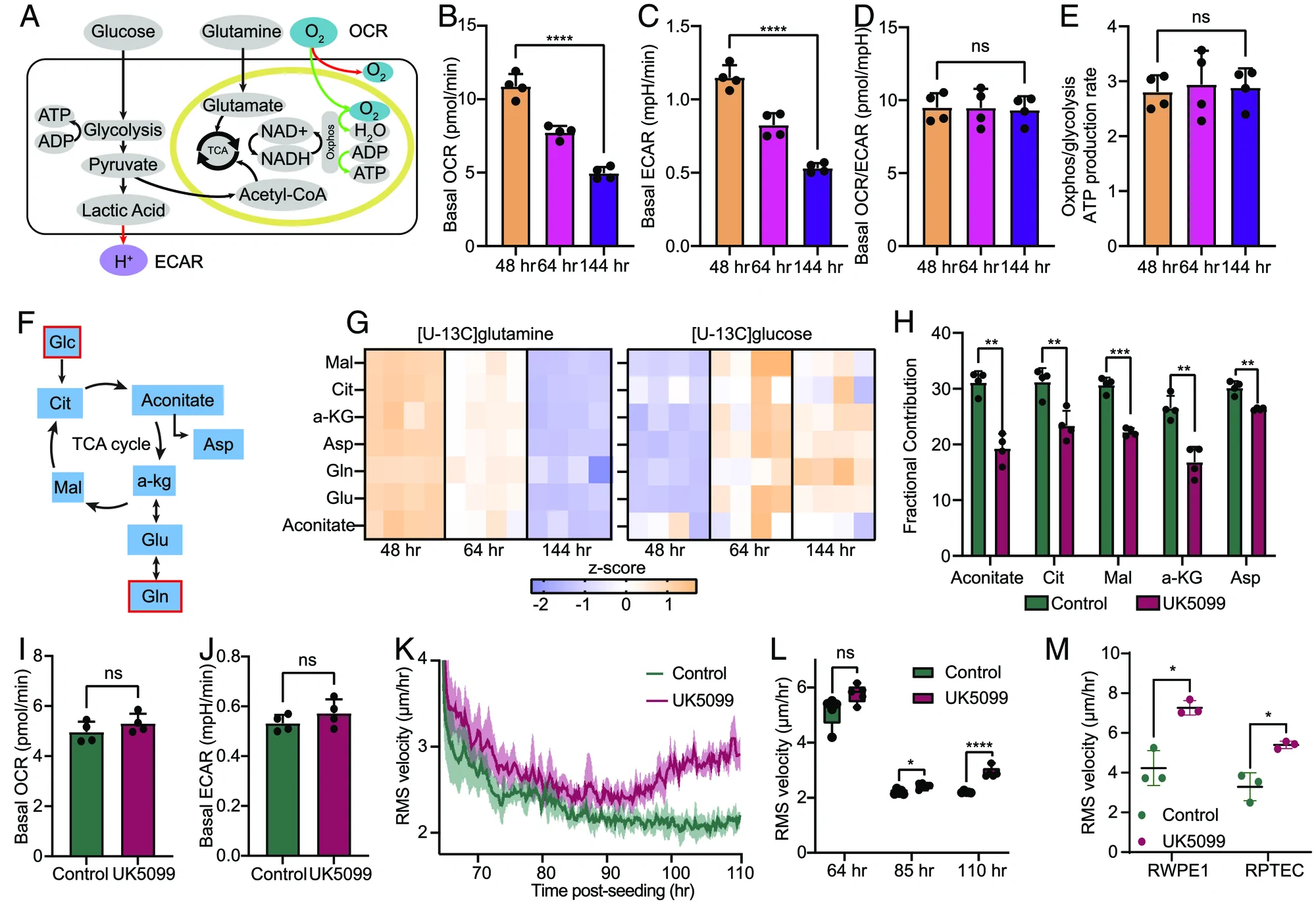
Pyruvate anaplerosis limits motility during epithelial crowding. (*A*) Seahorse assays were used to assess bioenergetic changes during crowding. OCR reflects oxidative phosphorylation supported largely by glucose- and glutamine-derived substrates, whereas ECAR primarily reports glycolytic proton efflux. (*B* and *C*) Both basal OCR and ECAR decrease by approximately 50% over the crowding time course. (*D*) The OCR/ECAR ratio remains stable, indicating a proportional downscaling of oxidative phosphorylation and glycolysis. (*E*) The relative ATP production from OXPHOS versus glycolysis also remains constant across time points. (*F*) Stable-isotope tracing with [U-^13^C]glutamine and [U-^13^C]glucose was performed to evaluate substrate routing into the TCA cycle. (*G*) Glutamine-derived carbon incorporation into TCA intermediates decreases over time, consistent with the global suppression of energy metabolism observed in Seahorse analyses, whereas glucose-derived carbon incorporation increases progressively. (*H*) Inhibition of mitochondrial pyruvate import with the MPC inhibitor UK5099 markedly reduces glucose-derived carbon labeling of TCA intermediates, confirming reduced mitochondrial pyruvate entry. (*I* and *J*) UK5099 treatment has minimal impact on basal OCR or ECAR, indicating preserved global respiration and glycolytic activity. (*K* and *L*) MPC inhibition delays the onset of the jamming transition and significantly increases collective cell motility, as reflected by elevated $v_{\mathrm{rms}}$ during crowding. (*M*) This increase in $v_{\mathrm{rms}}$ upon MPC inhibition is reproduced in RWPE-1 and RPTEC monolayers, demonstrating that pyruvate-dependent mitochondrial metabolism broadly limits epithelial motility during crowding. ns, *, **, *** and **** correspond to *P*-values > 0.05, ≤0.05, ≤0.01, ≤0.001, and ≤0.0001, respectively. Statistical comparisons were calculated using unpaired two-sided *t* tests. No bootstrapping was performed in any analysis. N = 4 independent biological replicates were used for *B*–*E* and *G*–*J*. N = 5 independent biological replicates were used for *K*–*L*. N = 3 independent biological replicates were used for *M*. Each replicate represents a separate well.

Following the identification of these early transcriptomic shifts, we sought to functionally characterize how cell crowding progression affects cellular energy production. We utilized a Seahorse XF Analyzer to perform real-time extracellular flux analysis, measuring the oxygen consumption rate (OCR) and extracellular acidification rate (ECAR) as indicators of mitochondrial oxidative phosphorylation and glycolytic activity, respectively (Fig. 2*A*).

Measurements were collected at 48, 64, and 144 h to capture the functional metabolic state during the onset of transcriptomic changes, the transitions in adhesion and YAP localization, and the attainment of a fully jammed state. We found that both basal OCR and ECAR decreased by approximately 50% over the course of the crowding process (Fig. 2 *B* and *C* and *SI Appendix*, Fig. S2*A*), while the OCR-to-ECAR ratio (Fig. 2*D*) and relative ATP production rates (Fig. 2*E*) remained approximately stable. This coordinated decline suggests a global downscaling of cellular energy metabolism during crowding, rather than a metabolic shift between oxidative phosphorylation and glycolysis. Consequently, overall energy production decreases as cells reach a jammed state (*SI Appendix*, Fig. S2*B*), indicating a significant reduction in metabolic activity under high-density conditions.

To further clarify the metabolic underpinnings of the energy downscaling observed in our Seahorse assays, we investigated how nutrient utilization is redistributed across major metabolic pathways. We performed stable-isotope tracing using uniformly labeled [U-^13^C]glutamine and [U-^13^C]glucose, the primary carbon sources fueling the tricarboxylic acid (TCA) cycle (Fig. 2*F*). Metabolomic analysis (*SI Appendix*, Fig. S2 *C* and *D*) revealed a progressive decline in the incorporation of glutamine-derived carbon into TCA cycle intermediates (Fig. 2*G* and *SI Appendix*, Fig. S2*E*). This reduction indicates a significant decrease in glutaminolysis-dependent anaplerosis during the jamming transition, consistent with the global suppression of energy metabolism identified via extracellular flux analysis. Together, these findings suggest a metabolic shift away from glutamine oxidation as TCA cycle replenishment transitions toward a more energetically quiescent state.

In striking contrast to the decline in glutamine utilization, [U-^13^C]glucose labeling revealed a progressive increase in the fractional incorporation of glucose-derived carbon into TCA cycle intermediates over time (Fig. 2*G* and *SI Appendix*, Fig. S2*F*), despite the overall decrease in energy production. Detailed isotopologue labeling patterns for these intermediates are provided in *SI Appendix*, Fig. S2 *G*–*L*. These tracer-derived data demonstrate that cells utilize proportionally more glucose as a carbon source for the TCA cycle during crowding, presenting a paradox in which glucose contribution increases while total ATP output declines.

To address the functional significance of this paradoxical increase in pyruvate anaplerosis, we inhibited mitochondrial pyruvate import using UK5099, a selective inhibitor of the mitochondrial pyruvate carrier (MPC). As expected, UK5099 treatment markedly reduced the incorporation of glucose-derived carbon into TCA cycle intermediates, confirming reduced mitochondrial pyruvate entry into the mitochondria (Fig. 2*H* and *SI Appendix*, Fig. S3*A*). Importantly, under this fully confluent condition when ATP demand is low, UK5099 does not alter basal OCR nor ECAR, and thus mitochondrial respiration and glycolytic activity (Fig. 2 *I* and *J* and *SI Appendix*, Fig. S3*B*). These results demonstrate that mitochondrial pyruvate restriction does not suppress global respiration, but rather specifically alters the metabolic substrate routing.

This inhibition of mitochondrial pyruvate import, however, led to a pronounced enhancement of collective cell motility during the crowding process (Fig. 2*K* and *SI Appendix*, Fig. S3*C* and Movie S2). Within 24 h of treatment (added at the 48 h time point), UK5099-treated cells displayed an approximately 40% increase in average migration speed relative to vehicle-treated controls at the 110 h mark (Fig. 2*L*). To validate the pharmacological findings, we generated a doxycycline-inducible CRISPRi MDCK line expressing two guide RNAs targeting canine MPC1. Doxycycline treatment substantially reduced MPC1 expression (*SI Appendix*, Fig. S3*D*) and significantly increased RMS velocity, recapitulating the effect of UK5099 (*SI Appendix*, Fig. S3*E*). Thus, genetic reduction of MPC1 independently supports a role for MPC-dependent mitochondrial pyruvate import in restraining epithelial motility. UK5099 also significantly increased RMS velocity in crowded monolayers of human renal proximal tubule epithelial cells (RPTECs) and RWPE-1 human prostate epithelial cells (Fig. 2*M* and Movie S3), indicating that MPC activity restrains crowding-associated motility across multiple epithelial models. We also ruled out glutamine depletion as the cause of this phenotype, as the increased motility persisted in cultures supplemented with GlutaMAX (*SI Appendix*, Fig. S3*F*). Together, these findings support a role for mitochondrial pyruvate anaplerosis in restraining cell motility during crowding.

To determine which branch of pyruvate metabolism regulates epithelial motility, we independently perturbed pyruvate production, mitochondrial import, and lactate conversion (*SI Appendix*, Fig. S3*G*). Inhibition of PKM2 with shikonin (0.5 μM), which restricts glycolytic pyruvate generation, significantly increased RMS velocity and produced a greater response than MPC inhibition with UK5099 (5 μM). In contrast, inhibition of LDH with galloflavin (20 μM), which restricts pyruvate-to-lactate conversion without directly blocking mitochondrial pyruvate import, did not increase motility (*SI Appendix*, Fig. S3*G*). The shared effect of PKM2 and MPC inhibition is reduced mitochondrial access to glycolytically derived pyruvate, whereas their expected effects on cytosolic pyruvate differ. These results, therefore, identify mitochondrial pyruvate availability, rather than cytosolic pyruvate accumulation, lactate production, or nonspecific glycolytic inhibition, as the metabolic feature most closely associated with epithelial fluidization.

### MPC Inhibition Promotes Coordinated Fluid-Like Dynamics in Epithelial Monolayers.

Building on the observation that UK5099 treatment enhances cell motility and delays the normal progression toward a jammed state, we examined how MPC inhibition impacts fundamental cell properties and epithelial identity. We found that MPC inhibition had negligible effects on cell proliferation and survival, as indicated by the proportion of EdU-positive and cleaved CASP3-positive cells, respectively. Furthermore, overall cell density remained unaffected by the treatment (*SI Appendix*, Fig. S4 *A*–*E*).

To characterize the morphological changes induced by UK5099, we first quantified cell aspect ratio and shape index (perimeter/$\sqrt{\mathrm{area}}$), two features associated with epithelial fluidization. UK5099 increased both measures, indicating pronounced changes in cell geometry (*SI Appendix*, Fig. S4 *F* and *G*). In aspect ratio–shape index space, the shift from control to UK5099-treated cells aligned with the trajectory induced by TGF-β1, a well-established driver of epithelial-to-mesenchymal transition (EMT) (*SI Appendix*, Fig. S4*H*). UK5099 also increased cell-boundary tortuosity, indicating greater irregularity of the cell perimeter (*SI Appendix*, Fig. S4*I*). Together, these morphological similarities raised the question of whether UK5099 induces EMT or produces an unjammed epithelial state.

We therefore examined the molecular characteristics of UK5099-treated cells. Despite the pronounced morphological changes, UK5099 did not significantly reduce total E-cadherin expression (*SI Appendix*, Fig. S5 *A*–*C*). The mean intensity of homophilically bound E-cadherin, representing its active junctional state, also remained unchanged relative to control cells (*SI Appendix*, Fig. S5 *D* and *E*). Thus, the UK5099-induced changes in cell geometry and perimeter irregularity occurred without detectable loss of E-cadherin-mediated epithelial adhesion.

We further quantified vimentin, junctional E-cadherin, and ZO-1 to distinguish unjamming from EMT. TGF-β1 increased vimentin expression and reduced junctional E-cadherin and ZO-1 intensities. In contrast, these markers remained similar between UK5099-treated and control cells (*SI Appendix*, Fig. S5 *F*–*I*). Consistent with these results, UK5099 did not activate detectable EMT-associated transcriptional programs (*SI Appendix*, Fig. S1 *F* and *G*). Together, these findings indicate that MPC inhibition produces morphological changes that overlap with those induced by TGF-β1 while preserving the epithelial markers examined, making the response more consistent with a mechanical jamming-to-unjamming transition than with activation of a conventional EMT program.

**Fig. 3.**
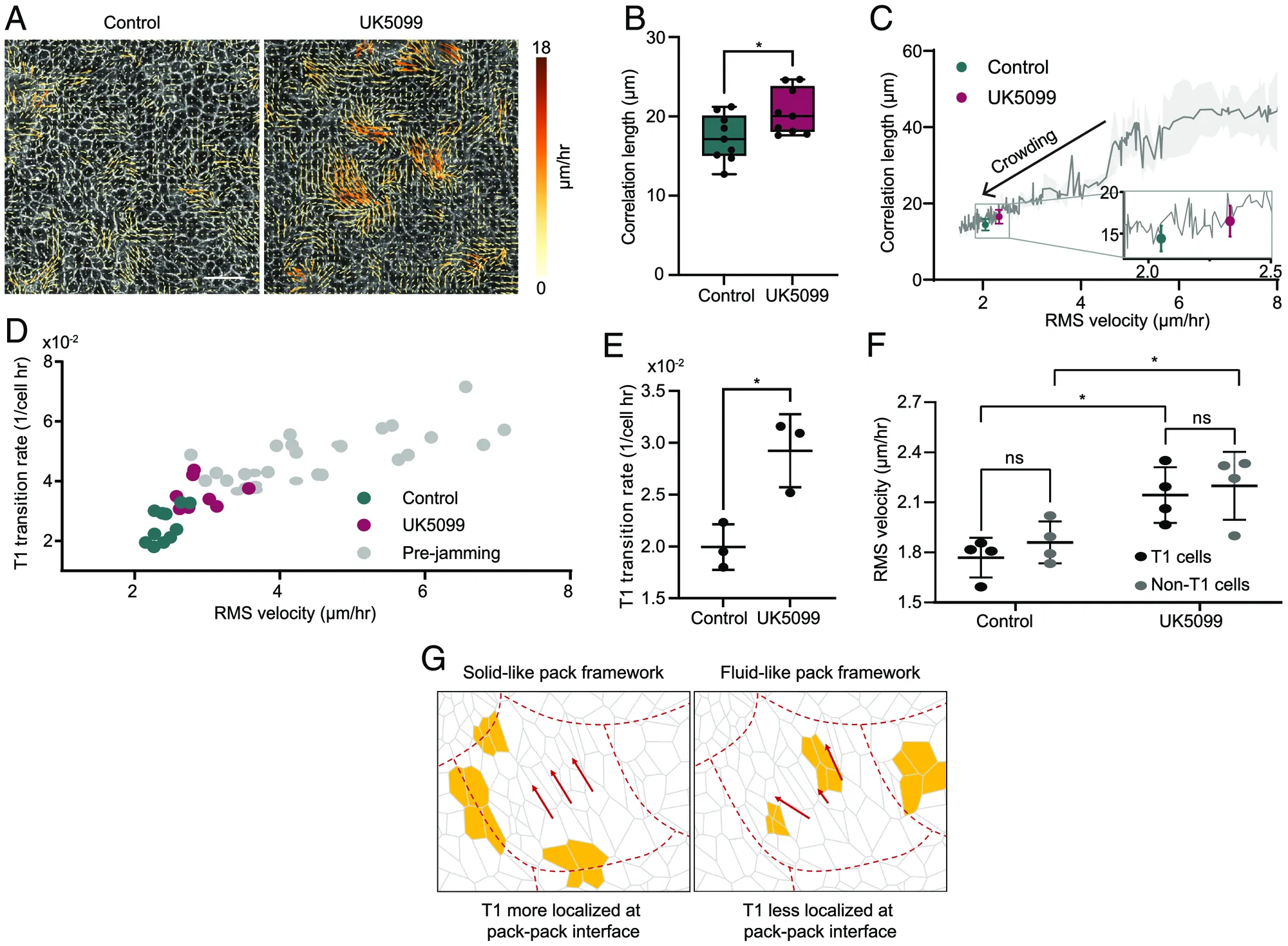
MPC inhibition induces a mechanically unjammed, fluid-like tissue state. (*A*) Representative velocity field maps from control and UK5099-treated monolayers at ∼100 h postseeding illustrate enhanced collective motion under MPC inhibition. (Scale bar, 50 μm.) (*B*) UK5099 produces an increase in the velocity correlation length $\xi_{v}$, indicating more long-range coordination of cell movement. n = 9 where n denotes the number of fields of view (FOVs) analyzed. (*C*) Plotting $\xi_{v}$ against $v_{\mathrm{rms}}$ shows that MPC inhibition induces a motility–coordination relationship characteristic of a prejamming, fluid-like state. The solid line represents the trajectory of untreated monolayers undergoing a jamming transition; shading denotes SD n = 9 where n denotes the FOVs analyzed. (*D*) The rate of T1 neighbor-exchange events exhibits an approximately linear dependence on $v_{\mathrm{rms}}$, consistent with increasing tissue fluidity. n = 9 where n denotes the number of FOVs analyzed. (*E*) UK5099 increases T1 transition frequency by ∼30 to 50%. n = 20 where n denotes the number of FOVs analyzed. (*F*) Instantaneous velocities of cells undergoing T1 transitions are comparable to non-T1 neighbors in both control and treated monolayers. n = 10 where n denotes the number of FOVs analyzed. (*G*) Together, these results suggest that MPC inhibition promotes coordinated yet fluid-like collective motion: correlation lengths increase with motility, while T1 events occur broadly throughout the tissue rather than being localized to pack interfaces. ns and * correspond to *P*-values > 0.05 and ≤ 0.05, respectively. Statistical comparisons were calculated using unpaired two-sided *t* tests. No bootstrapping was performed in any analysis. N = 4 biological replicates were used for *B*–*D* and *F*, and N = 3 biological replicates were used for *E*, with each replicate representing a separate well.

We next investigated whether UK5099 also enhances the coordination of motion within the epithelial sheet (Fig. 3*A*). The velocity correlation length, $\xi_{v}$, which measures the spatial persistence of velocity correlations across the tissue, serves as a quantitative indicator of collective dynamics: longer correlation lengths reflect greater mechanical coupling and coordinated movement, whereas shorter lengths characterize localized, jammed behavior (41). UK5099 treatment produced a modest but significant increase in correlation length (Fig. 3*B* and *SI Appendix*, Fig. S5*J*), indicating more cohesive, long-range coordination among neighboring cells. Within the jamming–unjamming framework, this shift signifies a transition toward a more fluid, prejamming state (Fig. 3*C*). The emergence of this coordinated, “pack-like” motion provided the basis for our subsequent analysis of collective topological rearrangements.

To evaluate how elevated coordination in cell motility modulates tissue architecture, we quantified T1 neighbor-exchange events (*SI Appendix*, Fig. S5*K* and Movies S4 and S5), a topological metric that signals increased fluidity approaching the unjamming point. In a rigid-pack model of collective motion, T1 transitions are typically confined to interfaces between cell packs and are expected to depend only weakly on mean cell speed (41). Using a simple scaling argument, we find that if T1 transitions occur primarily at cluster interfaces, the fraction of boundary cells scales as $f_{\mathrm{int}}∼1/L_{c}∼1/V_{\mathrm{rms}}$ (where $L_{c}$ is the cluster size). Since the relative speed between neighboring clusters scales as $V_{\mathrm{rel}}∼V_{\mathrm{rms}}$, the total T1 rate $R_{T1}∼f_{\mathrm{int}}V_{\mathrm{rel}}$ should theoretically remain independent of $V_{\mathrm{rms}}$. Contrary to this expectation, UK5099-treated monolayers exhibited approximately 30% higher T1 frequencies than untreated controls under identical crowding conditions (Fig. 3*E*). Although untreated prejamming and UK5099-treated monolayers both exhibited fluid-like behavior, their modes of movement differed. UK5099-treated monolayers displayed a higher T1 transition frequency than untreated prejamming monolayers (*SI Appendix*, Fig. S5*L*). This difference likely reflects their distinct cell densities: prejamming cells can migrate through coordinated colony expansion into available space without exchanging neighbors, whereas UK5099-treated cells remain densely packed and therefore rely more strongly on neighbor exchange to sustain movement. The increase in T1 transition rate for UK5099 treated cells (Fig. 3*E*) is consistent with a fluid-like UJT, further supported by the lack of global planar polarity (*SI Appendix*, Fig. S5 *M* and *N*) and elevated isotropic (*SI Appendix*, Fig. S5 *O* and *P*) and deviatoric (*SI Appendix*, Fig. S5 *Q* and *R*) strain rates. These results collectively indicate that UK5099 not only elevates collective cell movement but also promotes more frequent neighbor exchanges, reflecting an unjamming behavior distinct from simple pack translation and consistent with enhanced local fluidity across the tissue.

Finally, to link motility to topological dynamics at the single-cell level, we compared the instantaneous speeds of cells undergoing T1 transitions with those of nonrearranging neighbors. In both control and UK5099-treated conditions, T1 and non-T1 cells exhibited comparable velocities (Fig. 3*F*). Combined with the observed increases in velocity correlation length and T1 transition frequency, this result indicates that UK5099 promotes a partially coordinated yet fluid tissue state: cells move in fluid-like packs whose characteristic size increases with motility, while topological rearrangements remain widespread across the monolayer rather than being localized to pack interfaces (Fig. 3*G*). Altogether, these morphological, kinematic, and topological observations demonstrate that UK5099 induces a mechanical UJT characterized by preserved epithelial cohesion, enhanced collective coordination, and increased dynamic rearrangements within the cell layer.

### MPC Inhibition Promotes Motility through RhoA-Dependent Cytoskeletal Remodeling.

To elucidate how MPC inhibition drives increased cell motility, we next examined alterations in actin organization, as actin remodeling directly governs cell movement. To this end, we established an MDCK cell line expressing LifeAct-GFP to visualize filamentous actin (F-actin) in live cells (42). UK5099 treatment increased the number of stress fibers (Fig. 1 *A* and *B*). Notably, increased stress-fiber abundance was also observed in untreated subconfluent, prejamming cells relative to jammed cells (*SI Appendix*, Fig. S6 *A* and *B*), indicating that MPC inhibition recapitulates a cytoskeletal feature characteristic of the prejamming state. UK5099 did not alter the average thickness of individual stress fibers (*SI Appendix* Fig. S6 *C* and *D*).

**Fig. 4.**
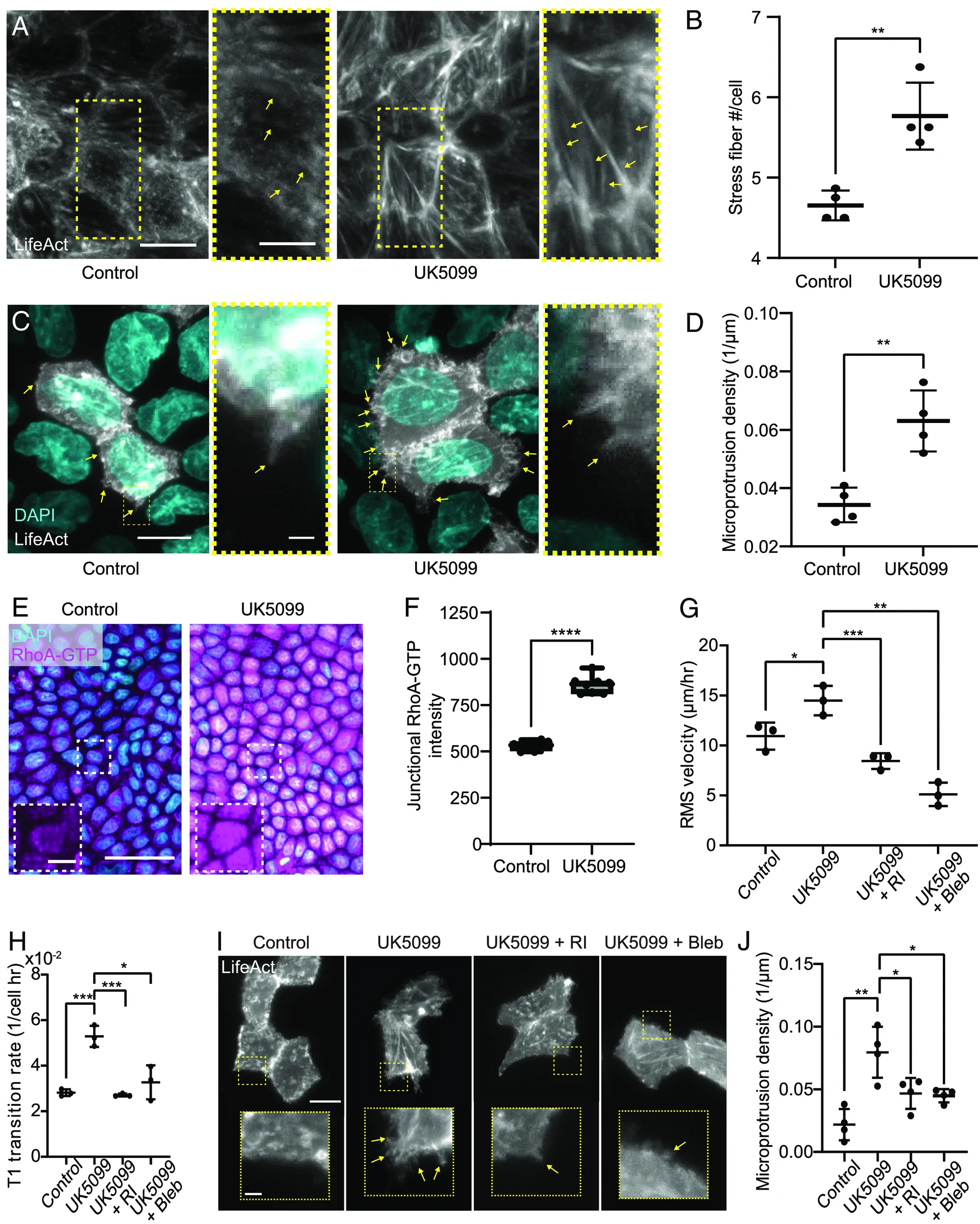
MPC inhibition enhances RhoA-dependent cytoskeletal remodeling to promote motility. (*A*) LifeAct-GFP imaging reveals an increase in the number of stress fibers in UK5099-treated monolayers. (Scale bar, 10 μm.) (*B*) Quantification confirms elevated stress fiber density. n = 16 where n represents the number of cells analyzed. (*C*) Sparse LifeAct-GFP labeling shows that MPC inhibition increases the frequency of cryptic lamellipodia (microprotrusions). (Scale bars, 10 μm and 1 μm, respectively.) (*D*) Microprotrusion density is significantly elevated in UK5099-treated cells. Measurements were performed with at least n = 25 where n represents the number of cells analyzed. (*E*) Immunostaining for active RhoA-GTP demonstrates higher signal intensity following MPC inhibition, indicating increased RhoA activity. (Scale bar, 50 μm.) (*F*) Junctional RhoA-GTP intensity is significantly elevated. n = 12 where n represents the number of FOVs analyzed. (*G*) Inhibiting downstream RhoA–ROCK signaling (Y-27632) or myosin II activity (blebbistatin) abolishes the UK5099-induced increase in cell motility. (*H*) The UK5099-driven rise in T1 neighbor-exchange events is similarly suppressed by ROCK or myosin II inhibition. n = 10 where n represents the number of FOVs analyzed. (*I* and *J*) Both ROCK and myosin II inhibition markedly reduce microprotrusion formation and density. Measurements were performed with at least n = 25 where n represents the number of cells analyzed. (Scale bars, 10 μm and 1 μm, respectively.) *, **, *** and **** correspond to *P*-values ≤ 0.05, ≤ 0.01, ≤ 0.001, and ≤ 0.0001, respectively. Statistical comparisons were calculated using unpaired two-sided *t* tests. No bootstrapping was performed in any analysis. N = 4 independent biological replicates were used for *B*, *D*, and *J*. N = 8 independent biological replicates were used for *F*. N = 3 independent biological replicates were used for *G* and *H*, with each replicate representing a separate well.

Since increased actin assembly often correlates with greater actin-driven protrusive activity, we quantified the density of cryptic lamellipodia—thin, actin-rich protrusions that extend beneath neighboring cells to mediate traction transmission during collective migration. Because these fine structures are easily obscured in confluent, uniformly labeled monolayers, we employed a sparse-labeling strategy, mixing a small fraction of LifeAct-GFP-expressing cells with unlabeled cells (*SI Appendix*, Fig. S6*E*). Using this system, we found that UK5099-treated cells displayed a significantly higher frequency of cryptic lamellipodia (Fig. 4 *C* and *D*), though the average protrusion length remained unchanged (*SI Appendix*, Fig. S6*F*). These results are consistent with elevated actin turnover and enhanced coordination of collective motility following MPC inhibition.

We next investigated whether the enhanced actin remodeling observed in UK5099-treated cells was driven by elevated activity of RhoA, a key regulator of actin assembly. Immunostaining for active GTP-bound RhoA using a conformation-specific antibody revealed an approximately 60% increase in signal intensity, both within individual cells and along cell–cell junctions, when MPC function was blocked (Fig. 4 *E* and *F*). The increase in RhoA activity was accompanied by elevated total RhoA levels (*SI Appendix*, Fig. S6 *G* and *H*) and a moderate increase in Rac1-GTP intensity (*SI Appendix*, Fig. S6 *I* and *J*). Elevated RhoA-GTP and Rac1-GTP signals were also observed in untreated subconfluent, prejamming cells relative to jammed cells (*SI Appendix*, Fig. S6 *K*–*N*), further linking the UK5099-induced phenotype to the prejamming state.

To test the functional significance of RhoA in these phenotypes, we simultaneously inhibited MPC and ROCK, a crucial downstream effector of RhoA that promotes actin polymerization and myosin II activation. Treatment with the ROCK inhibitor Y-27632 significantly decreased cell motility (Fig. 4*G*), the frequency of T1 transitions (Fig. 4*H*), and the density of periph eral microprotrusions (Fig. 4 *I* and *J*). Consistent with these results, inhibition of myosin II using blebbistatin similarly abolished these activities (Fig. 4*G*–*J*). Together, these findings identify RhoA and actomyosin as central mediators of the UK5099-induced increase in actin remodeling and cell motility. Mitochondrial pyruvate restriction thus enhances RhoA-ROCK-dependent actin assembly, driving cytoskeletal reorganization and motility during epithelial crowding.

### MPC Inhibition Enhances Endocytic Remodeling to Drive Epithelial Cell Motility.

Live-cell imaging of LifeAct-GFP-expressing epithelial monolayers revealed that UK5099-treated cells frequently exhibited dynamic membrane ruffling followed by the formation of circular enclosed structures (Movies S6 and S7). These features are characteristic of macropinocytosis, an actin-driven, clathrin-independent form of endocytosis that enables the internalization of extracellular fluid and serves as an auxiliary nutrient acquisition pathway (43).

**Fig. 5.**
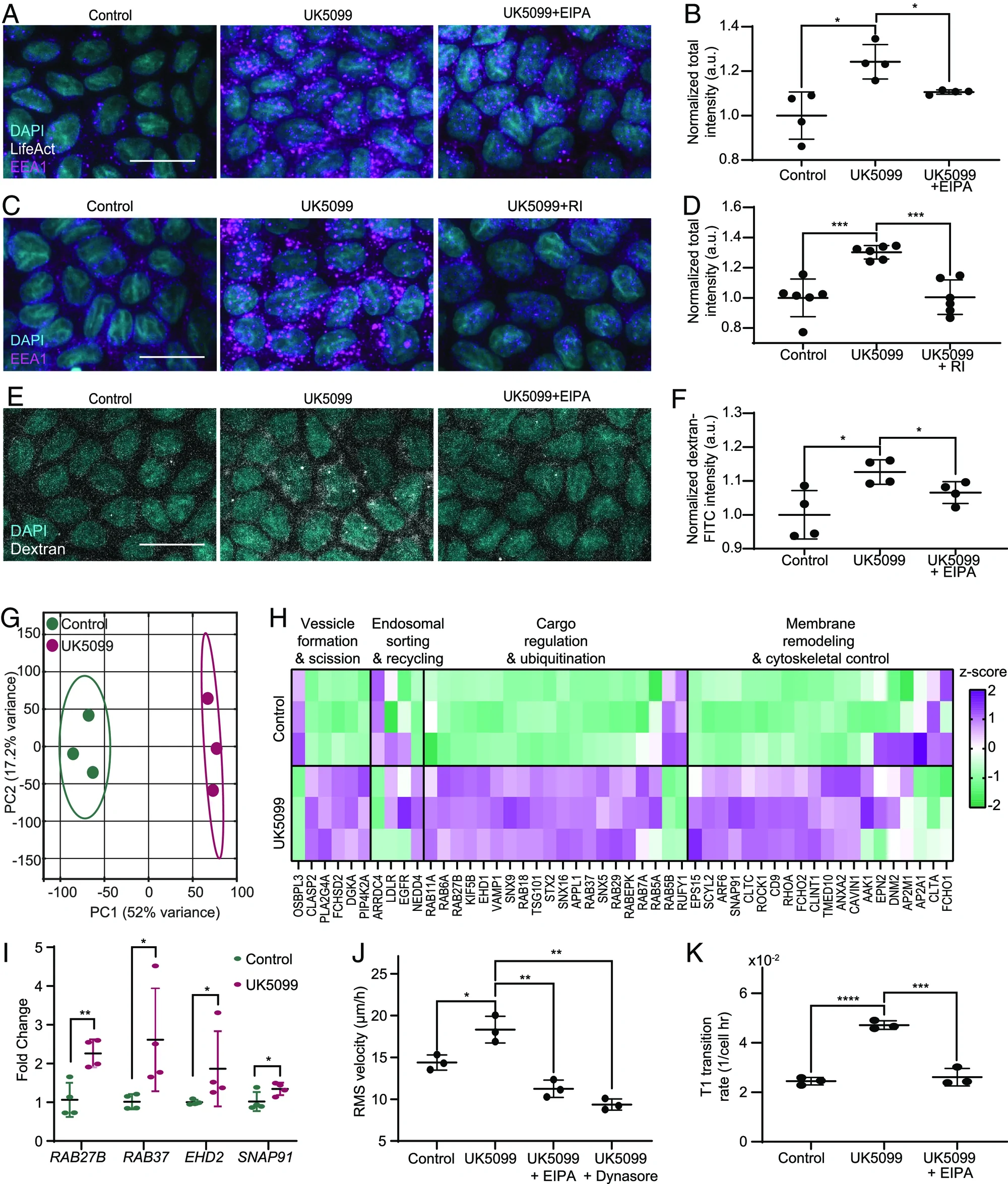
MPC inhibition enhances macropinocytosis to drive epithelial motility. (*A* and *B*) Representative immunofluorescent images (*A*) and quantification (*B*) of the early endosome marker EEA1. Treatment with UK5099 increases EEA1 intensity, which is reversed by the Na^+^/H^+^ exchange inhibitor EIPA. Measurements were conducted with at least n = 200 cells. (*C* and *D*) Representative images (*C*) and quantification (*D*) of EEA1 staining showing that Rho-kinase inhibition (Y-27632) prevents the UK5099-induced expansion of early endosomes. (Scale bar, 20 μm for *A*, *C*, and *E*.) Measurements were conducted with at least n = 200 cells. (*E* and *F*) Representative images (*E*) and quantification (*F*) of Dextran-FITC uptake assays, indicating that UK5099 enhances bulk fluid-phase endocytosis (macropinocytosis) in an EIPA-sensitive manner. Measurements were conducted with at least n = 200 cells. (*G*) PCA plot of RNA-seq data showing distinct transcriptional separation between control and MPC-inhibited (UK5099) samples. (*H*) Heatmap displaying the coordinated upregulation of major endocytosis-associated genes in UK5099-treated cells. (*I*) qPCR validation confirms the upregulation of key vesicular trafficking genes (e.g., *RAB27B*, *SNAP91*). (*J*) Functional analysis showing that blocking macropinocytosis with EIPA or endocytosis with Dynasore abolishes the UK5099-induced increase in cell motility. (*K*) Inhibition of macropinocytosis with EIPA reduces the UK5099-induced increase in the T1 neighbor-exchange rate. n = 10 where n represents the number of FOVs analyzed. *, **, *** and **** correspond to *P*-values ≤ 0.05, ≤ 0.01, ≤ 0.001, and ≤ 0.0001, respectively. Statistical comparisons were calculated using unpaired two-sided *t* tests. No bootstrapping was performed in any analysis. N = 4 independent biological replicates were used for *B*, *F*, and *I*. N = 6 independent biological replicates were used for *D*. N = 3 independent biological replicates were used for all other analyses, with each replicate representing a separate well.

To investigate this link, we quantified EEA1-positive early endosomes and found that MPC inhibition increased their density (Fig. 5 *A* and *B*). This increase can be fully reversed by EIPA [5-(N-ethyl-N-isopropyl)amiloride], a Na^+^/H^+^-exchanger inhibitor commonly used to suppress macropinocytosis. We further found that the expansion of early endosomes was reversed by Y-27632 treatment (Fig. 5 *C* and *D*), suggesting that RhoA activity mediates this endocytic upregulation. In contrast, inhibiting endocytosis had negligible effects on cytoskeletal organization, such as stress fiber density (*SI Appendix*, Fig. S7 *A* and *B*) and microprotrusion density (*SI Appendix*, Fig. S7 *C* and *D*). Dextran uptake assays revealed enhanced bulk fluid-phase endocytosis in UK5099-treated cells (Fig. 5 *E* and *F*), further supporting an overall increase in macropinocytic capacity.

To uncover the transcriptional basis of this enhanced endocytic activity, we performed bulk RNA-seq analysis, which revealed a clear segregation between control and UK5099-treated samples in principal component space. This distinct clustering reflects global transcriptional reprogramming following metabolic perturbation (Fig. 5*G*). Our analysis specifically highlighted an upregulation of RhoA-associated genes (*SI Appendix*, Fig. S7*E*) and confirmed that EMT activation was absent (*SI Appendix*, Fig. S7*F*). Furthermore, analysis of endocytosis-related transcripts showed a coordinated upregulation in UK5099-treated cells, as visualized by z-scored expression heatmaps (Fig. 5*H*). Several key regulators of vesicular trafficking, including *RAB27B*, *RAB37*, *EHD2*, and *SNAP91*, were significantly upregulated, a result we validated via quantitative PCR (Fig. 5*I*). These findings indicate that MPC inhibition fundamentally upregulates the expression of the endocytic machinery.

Finally, to test whether this increased macropinocytic activity is functionally required for the observed motility phenotype, we treated cells with EIPA in the presence of UK5099. Inhibition of macropinocytosis completely abolished the UK5099-induced increase in cell motility (Fig. 5*J* and Movie S8) and the T1 transition rate (Fig. 5*K*), demonstrating that endocytic remodeling is essential for the migratory response. Critically, this macropinocytosis-driven motility was also observed in the naturally motile prejamming state, as untreated subconfluent cells also exhibited a higher abundance of EEA1-positive early endosomes than jammed control cells (*SI Appendix*, Fig. S7 *G* and *H*). Moreover, EIPA treatment reduced cell motility at the crowded, prejamming stage (*SI Appendix*, Fig. S7*I*), but not in an earlier stage when cells were still sparsely distributed. These findings suggest that macropinocytosis becomes important for sustaining cell motility as epithelial monolayers become crowded and support the idea that MPC inhibition maintains this endocytic feature of the prejamming state.

Together, these findings establish that MPC inhibition enhances macropinocytosis, which in turn promotes collective epithelial cell motility. These findings define a signaling axis that links altered mitochondrial metabolism to the mechanical and behavioral reprogramming of epithelial tissues through endocytic control.

## Discussion

Despite the collection of transcriptional data related to cell confluence (44), its temporal evolutions and biological consequences were previously unknown. Our findings demonstrate that metabolic programs actively modulate epithelial jamming transitions, revealing a regulatory dimension that extends beyond geometric constraints and previously recognized mechanical influences. While unjamming and collective migration have been associated with increased glycolytic and oxidative metabolism (34, 45) and ATP-intensive biosynthetic demands (46, 47), the causal influence of specific metabolic pathways on cellular jamming has remained largely untested. Here, we address this gap by showing that metabolic reprogramming regulates jamming in two key ways. First, metabolic remodeling during crowding is not merely a byproduct of jamming; rather, early in the crowding process, epithelial cells adopt a pyruvate-anaplerotic program that subsequently restrains motility. Perturbing this program is sufficient to enhance cell movement and delay jamming without inducing a mesenchymal fate. Second, this regulatory mode is independent of overall energy production, as restricting mitochondrial pyruvate import alters motility despite minimal effects on oxidative phosphorylation or total ATP generation. Together, these results identify MPC-dependent pyruvate anaplerosis as a key regulator of epithelial jamming, distinct from the energy-producing glycolytic states associated with mechanically induced unjamming (34).

We further show that disrupting mitochondrial pyruvate import induces a fluid-like epithelial state that aligns with the hallmark features of unjamming. MPC inhibition increased velocity-correlation lengths and produced coordinated, pack-like flows characteristic of fluidized epithelia (22, 28, 48, 49). This inhibition also elevated the frequency of T1 transitions, consistent with theories identifying T1 rearrangements as a primary route to tissue fluidization (50, 51). The comparable speeds observed between T1-engaged and non-T1-engaged cells further support a model in which fluidity emerges from collective dynamics rather than from a subset of highly motile cells within relatively rigid packs (41). The reproducible increase in motility following inhibition of pyruvate metabolism indicates that epithelial dynamics are sensitive to mitochondrial pyruvate utilization. This conclusion is strengthened by the concordant effects of inducible MPC1 knockdown, UK5099 treatment, and upstream restriction of pyruvate generation. Together, these findings identify MPC-dependent mitochondrial pyruvate utilization as an important metabolic regulator of epithelial fluidization during crowding, within the broader network of processes governing the jamming transition.

Shape-based distinctions between unjamming and EMT appear to be context-dependent. In contrast to the morphological separation reported in primary airway epithelia (22), UK5099- and TGF-β1-treated cells followed similar shape index–aspect ratio trajectories, indicating that these measures alone cannot classify the transition. However, UK5099 induced fluid-like collective dynamics without reducing junctional E-cadherin or ZO-1, increasing vimentin expression, or activating EMT-associated transcriptional programs. Thus, under the conditions examined, UK5099-induced fluidization is more consistent with mechanical unjamming than with a conventional EMT program, although transient or limited EMT-like changes cannot be excluded.

This fluidization phenotype was accompanied by RhoA-actomyosin remodeling, with ROCK or myosin II inhibition suppressing the associated motility and rearrangement phenotypes. The differential responses to UK5099, shikonin, and galloflavin further identify mitochondrial pyruvate availability, rather than cytosolic pyruvate accumulation or lactate production, as the metabolic variable associated with fluidization. We hypothesize that the stronger shikonin phenotype reflects the broader consequences of restricting pyruvate generation upstream of both mitochondrial import and lactate production, including altered carbon routing and redox signaling. However, because pyruvate flux was not measured following shikonin treatment and PKM2-independent effects cannot be excluded, this interpretation remains tentative. Our results therefore support a functional, rather than direct molecular, connection between mitochondrial pyruvate metabolism and actomyosin organization. Future isotope-tracing and metabolic-rescue experiments will be needed to identify the intermediate signal and determine whether increasing mitochondrial pyruvate utilization is sufficient to promote jamming at lower cell densities.

Finally, our results demonstrate that increased endocytosis acts downstream of the MPC-RhoA–actomyosin axis to sustain cell motility in crowded epithelia. This echoes prior work showing that enhanced endocytosis promotes epithelial fluidization (27, 52, 53); in particular, RAB5A-induced early endosome formation and macropinocytosis are known to unjam crowded epithelia (28).

Our study reveals mitochondrial pyruvate utilization as a key upstream regulator of this endocytosis-driven unjamming response. This finding establishes a signaling axis linking cellular crowding to pyruvate anaplerosis, ROCK-dependent actin remodeling, and the endocytic control of epithelial mechanics. Although MPC inhibition increased motility across canine and human epithelial models of kidney and prostate origin, the downstream RhoA/Rac1-dependent cytoskeletal and endocytic mechanisms were characterized primarily in MDCK cells. Whether the complete mechanistic pathway is conserved across other epithelial jamming transitions remains to be determined. Ultimately, this work unveils a previously unappreciated metabolic mechanism through which epithelial cells regulate endocytic activity to control the jamming–unjamming transition during tissue crowding.

## Materials and Methods

### Cell Culture and Drug Treatment.

MDCK cells were cultured in Minimum Essential Medium Alpha (MEM-α; Fisher Scientific, 12561-056) supplemented with 10% fetal bovine serum (FBS; Fisher Scientific, 12662-029) and 1% Penicillin–Streptomycin (Fisher Scientific, 15140-122). Cells were maintained at 37 Â°C in a humidified incubator with 5% CO_2_. For routine maintenance, cells were passaged at approximately 80% confluence using Trypsin–EDTA solution (Fisher Scientific, 25300-054). Experimental cultures were seeded at a density of 30,000 cells/cm^2^. For comparisons between epithelial density states, untreated prejamming MDCK monolayers were analyzed at approximately 90% confluence, 50 h after seeding. hTERT-immortalized renal proximal tubule epithelial cells (RPTECs) were cultured in ATCC-formulated DMEM:F-12 supplemented with the hTERT RPTEC Growth Kit to final concentrations of 5 pM triiodo-L-thyronine, 10 ng/mL recombinant human EGF, 3.5 μg/mL ascorbic acid, 5 μg/mL human transferrin, 5 μg/mL insulin, 25 ng/mL prostaglandin E1, 25 ng/mL hydrocortisone, 8.65 ng/mL sodium selenite, and 1.2 mg/mL sodium bicarbonate. The medium was additionally supplemented with 0.1 mg/mL G418. RWPE-1 cells were cultured in Keratinocyte Serum-Free Medium (K-SFM; catalog no. 17005-042) supplemented with 0.05 mg/mL bovine pituitary extract and 5 ng/mL EGF.

For MPC inhibition, cells were treated with 2 or 5 μM UK5099 (Selleckchem, S5317). For the EMT-inducing reference condition, MDCK cells were cultured in medium containing 1% FBS and treated with 10 ng/mL recombinant human TGF-β1 (PeproTech, 100-21-50UG) for 24 h. To perturb other branches of pyruvate metabolism, cells were treated with 0.5 μM shikonin (MedChemExpress, HY-N0822) or 20 μM galloflavin (MedChemExpress, HY-W040118), beginning 24 h after seeding and continuing for 3 d before imaging. For inhibition of Rho-associated kinase and nonmuscle myosin II, cells were treated with 10 μM Y-27632 or 1 μM blebbistatin, respectively (54–56). Y-27632 and blebbistatin treatments were initiated 24 and 48 h before analysis, respectively. These concentrations were optimized to achieve their on-target effects while minimizing cytotoxicity or induction of apoptosis. Unless otherwise specified, pharmacological treatments were initiated near the onset of confluence, approximately 48 to 64 h after seeding.

### Generation of Stable Cell Lines.

To visualize F-actin dynamics, we generated a stable MDCK cell line expressing LifeAct-14–eGFP. Lentiviral particles were produced in HEK293T cells using the LifeAct-14 transfer plasmid (Addgene, 158750) and subsequently used to transduce parental MDCK cells. Following transduction, cells were selected with 15 μg/mL blasticidin for 4 d to establish a homogeneous population with stable LifeAct-14–eGFP expression. Stable integrants were expanded and validated via fluorescence microscopy, which confirmed robust and consistent labeling of cortical F-actin. For experiments requiring the visualization of subcellular structures in confluent monolayers, these LifeAct-GFP-expressing cells were mixed with unlabeled parental MDCK cells at a 1:20 ratio to achieve sparse labeling.

A doxycycline-inducible MPC1 CRISPR-interference (CRISPRi) MDCK cell line was generated using a sequential dual-vector system. MDCK cells were first cotransfected with the piggyBac vector PB-TRE-dCas9-Zim3-MeCP2 (Addgene plasmid #220143) and a Super PiggyBac Transposase helper plasmid. Cells were selected with 800 μg/mL hygromycin to establish a stable polyclonal parental population harboring the TRE3G-controlled dCas9-Zim3-MeCP2 repressor. These cells were subsequently transduced, in the presence of 8 μg/mL polybrene, with a custom lentiviral vector encoding two guide RNAs targeting canine MPC1 (VectorBuilder). Following selection with 10 μg/mL puromycin, the resulting stable polyclonal CRISPRi cells were treated with 1 μg/mL doxycycline for 72 h to induce MPC1 transcriptional repression before imaging. MPC1 depletion was confirmed by western blotting.

### Experimental Procedures.

Detailed experimental procedures are provided in *SI Appendix*.

## Supplementary Material

Appendix 01 (PDF)

Movie S1. Supplemental Video 1: Time-lapse phase-contrast microscopy showing MDCK cells undergoing jamming and the associated arrest of cell motility. The video corresponds to the root-mean-square (RMS) velocity quantification shown in Figure 1B.

Movie S2. Supplemental Video 2: MPC inhibition enhances collective cell motility. Representative time-lapse phase-contrast microscopy (top) and corresponding root-mean-square (RMS) velocity fields (bottom) of cells treated with the MPC inhibitor UK5099. Arrow plots indicate the magnitude and direction of the velocity field, demonstrating increased motility compared to control conditions.

Movie S3. Supplemental Video 3: MPC inhibition enhances motility in RPTEC and RWPE1 cells. Representative time-lapse phase-contrast microscopy of RPTEC (top) and RWPE1 (bottom) cells treated with the MPC inhibitor UK5099. The videos correspond to the root-mean-square (RMS) velocity quantification in Figure 2M, demonstrating increased motility compared to control conditions.

Movie S4. Supplemental Video 4: Representative T1 transition in control cells. Time-lapse imaging capturing a neighbor exchange (T1 transition) within a control epithelial monolayer. Green contours highlight the "arriving" cell pair (gaining contact), while red contours highlight the "departing" cell pair (losing contact).

Movie S5. Supplemental Video 5: Representative T1 transition in UK5099-treated cells. Time-lapse imaging capturing a neighbor exchange (T1 transition) following MPC inhibition. Green contours highlight the arriving cell pair (gaining contact), and red contours highlight the departing cell pair (losing contact).

Movie S6. Supplemental Video 6: F-actin dynamics and baseline membrane ruffling in control cells. Time-lapse fluorescence microscopy of a sparsely labeled LifeAct-GFP cell, showing characteristic baseline mem brane ruffling and cytoskeleton organization in the control group.

Movie S7. Supplemental Video 7: Enhanced membrane ruffling following UK5099 treatment. Time-lapse fluorescence microscopy of a sparsely labeled LifeAct-GFP cell, showing accelerated F-actin polymerization and enhanced membrane ruffling dynamics induced by MPC inhibition.

Movie S8. Supplemental Video 8: Inhibition of macropinocytosis or endocytosis reverses the increase in cell motility induced by MPC inhibition. Macropinocytosis was inhibited with EIPA, and endocytosis was inhibited with Dynasore. Representative time-lapse phase-contrast microscopy of control (top left), UK5099-treated (top right), UK5099 + EIPA-treated (bottom left), and UK5099 + Dynasore-treated (bottom right) cells. The videos correspond to the root-mean-square (RMS) velocity quantification shown in Figure 5J and demonstrate that inhibition of either macropinocytosis or endocytosis abolishes the UK5099-induced increase in cell motility.

## Data Availability

RNA-Seq data have been deposited in Gene Expression Omnibus (GEO) (GSE318427 and GSE318423) (57, 58).
