# Supplementary material for: Cell jamming transition is regulated by mitochondrial pyruvate transport and endocytosis
Source: Proc Natl Acad Sci U S A. 2026 Oct 7;123(41):e2617216123. doi: 10.1073/pnas.2617216123 (PMC13663687; doi:10.1073/pnas.2617216123)
Supplement: Supplementary file 1 [file pnas.2617216123.sapp.pdf]

Supporting Information for

Cell jamming transition is regulated by mitochondrial pyruvate transport and endocytosis

Alexandra Bermudez†, Zoe Latham†, Johnny Diaz, Weihong Yan, Jerry Chen, Dapeng Bi, Andrew Goldstein, Jimmy K Hu*, Neil Y.C. Lin*

Jimmy K Hu and Neil Y.C. Lin.
E-mail: jkhu@g.ucla.edu and neillin@g.ucla.edu

This PDF file includes:

Supporting text
Figs. S1 to S7
Legends for Movies S1 to S8
SI References

Other supporting materials for this manuscript include the following:

Movies S1 to S8

Supporting Information Text

Materials and Methods

RNA-seq Analysis. Total RNA was extracted from cells using the Zymo RNA Extraction Kit (R2072) according to the manufacturer's protocol. RNA sequencing was performed on a single lane of an Illumina NovaSeq S4 flow cell (200-cycle kit), achieving an average read depth of approximately 44 million reads per sample. Raw RNA-seq reads were first subjected to quality assessment using FastQC. Adapter sequences and low-quality bases were trimmed with Cutadapt (1). The cleaned reads were then aligned to the domestic dog reference genome (CanFam4) using STAR (version 2.7.10a), an ultrafast RNA-seq aligner (2). Gene annotations were derived from the NCBI RefSeq All dataset, obtained from the UCSC Genome Browser (https://genome.ucsc.edu). Gene-level read counts generated by STAR were normalized and analyzed for differential expression using the `limma` package in R (3). For the GSEA analysis, differential expression between T5 (64 h post-seeding) and T1 (24 h post-seeding) was analyzed using limma. All six samples, three independent biological replicates per time point, were retained as separate samples in this group-level comparison and were not pooled. Genes were ranked according to the limma differential-expression results from the T5-versus-T1 contrast, and GSEA was performed on this ranked list using curated gene sets from the Molecular Signatures Database (MSigDB). T1 and T5 were selected to capture transcriptional changes during early crowding, before the inflection point observed in Fig. 1G. The UK5099-versus-control RNA-seq dataset was analyzed using the same differential-expression and enrichment workflow. For the E-cadherin-loss analysis, we used the predefined $ONDER\_CDH1\_TARGETS\_1\_UP$ gene set, which comprises genes upregulated in immortalized, nontransformed human mammary epithelial (HMLE) cells expressing dominant-negative E-cadherin(4). The complete predefined gene set was used for enrichment analysis. Representative genes were selected from this set for visualization based on published associations with E-cadherin loss and epithelial–mesenchymal transition; this selection did not affect the enrichment analysis. The RNA-seq datasets generated in this study have been deposited in the NCBI Gene Expression Omnibus (GEO) and are publicly accessible. The time-course and UK5099 datasets are available under accession numbers GSE318423 and GSE318427, respectively, through the NCBI GEO portal (https://www.ncbi.nlm.nih.gov/geo/).

Immunostaining. Cells were cultured in 4-chamber Ibidi slides (Ibidi, 80426) and fixed with 10% neutral-buffered formalin containing 0.03% Eosin (Sigma-Aldrich, F5304-4L) for 10 minutes at room temperature. Permeabilization and blocking were performed for 30 minutes in PBS containing calcium and magnesium (Fisher Scientific, 14040-133), supplemented with 2% donkey serum (Fisher Scientific, D9663-10ML) and 0.25% Triton X-100. Following three washes with PBS, cells were incubated for 30 minutes with the following primary antibodies using a 1:500 dilution: E-cadherin (Abcam, ab11512), YAP (Novus Biologicals, NB110-58358), RhoA-GTP (NewEast Biosciences, 26904), RhoA (Cell Signaling Technology, 2117T), Rac-1 GTP (NewEast Biosciences, 26903), EEA1 (BD Biosciences, 610456), ZO-1 (Cell Signaling Technology, 15652T), and vimentin (Invitrogen, MA5-11883). After washing three times with PBS, cells were incubated for 30 minutes at room temperature with secondary antibodies, anti-mouse Alexa Fluor 488 or anti-rabbit Alexa Fluor 647 (Invitrogen, A21042; Cell Signaling Technology, 4414S, respectively), together with DAPI (Abcam, ab228549) and either phalloidin (Invitrogen, A12379) or actin stain (Invitrogen, R37110). Dilutions were 1:1000 for secondary antibodies and DAPI, and 1:400 for phalloidin.

Microscopy and Image Analysis. Fluorescence images were acquired using either confocal or widefield microscopy systems. Confocal imaging was performed using a Rescan Confocal Microscope (RCM1) integrated with a Nikon Eclipse Ti-E (NIS-Elements software) or an NL5+ system mounted on a Nikon Ni-E or Zeiss AXIO Observer Z1 (Micro-Manager software). Widefield imaging was conducted via an Etaluma LS720 (LumaView 720/600-Series software) or an ECHO REVOLUTION microscope.

For confocal acquisitions, we utilized 20× WI (0.95 NA), 20× (0.75 NA), 40× oil (1.30 NA), and 63× oil (1.40 NA) objectives. Widefield imaging employed a 20× (0.40 NA) objective on the Etaluma system and a 20× (0.75 NA) objective on the ECHO REVOLUTION microscope. Imaging parameters, including laser power and exposure times, were maintained consistently across all experimental conditions to ensure comparability. For intensity quantification, z-projected images were generated from z-stack acquisitions with a 1.5 μm step size.

All image analyses were performed using `ImageJ/Fiji` (version 2.1.0/1.53c) or `MATLAB` (version R2023a). For junctional E-cadherin quantification, we used `Fiji` to measure E-cadherin immunofluorescence junctional intensity which was then normalized to the cytoplasmic background intensity (Appendix Figure 1A). To determine the YAP cytoplasmic-to-nuclear intensity ratio, we quantified the fluorescence intensities within the nuclear and cytoplasmic compartments independently after DAPI and actin segmentation, respectively, to delineate each region. To quantify junctional RhoA-GTP, image analysis was performed using MATLAB in which Phalloidin immunofluorescence images were used to define the junctional ROI. A binary mask was then generated by applying a global threshold to the Phalloidin channel to isolate the filamentous network. This mask was then superimposed onto the corresponding RhoA-GTP channel. Colocalization was quantified by calculating the fluorescence intensity of the RhoA signal within the Phalloidin-masked area. EEA1 and Dextran-FITC levels were quantified by measuring the integrated density per cell normalized to the control group.

Time-lapse Collective Motility and Live Imaging. To characterize collective cell dynamics, time-lapse imaging was performed under controlled environmental conditions. For cell motility quantification, MDCK monolayers were maintained in a humidified incubator at 37 °C and 5% CO_2 and imaged using the Etaluma LS720. Phase-contrast images were acquired at 15-minute intervals for up to 144 h (6 days) using a 20× Long Working Distance (LWD) U Plan Fluorite objective (NA=0.5, WD=2.1 mm,

CGT=0.17 mm; Olympus UPLFLN20X). Culture media were refreshed daily during long-term acquisitions. High-resolution characterization of cytoskeletal dynamics in LifeAct-GFP-expressing cells was performed on the NL5+ confocal system using a 63× oil (1.40 NA) objective with acquisition intervals ranging from 1 to 15 minutes.

Quantification of Kinematics and Topological Rearrangements. Cellular velocity fields were quantified using Particle Image Velocimetry (PIV) analysis in `MATLAB`. The root-mean-square cell velocity (V_{rms}) was calculated as: $V_{rms} = \sqrt{\langle v^2 \rangle}$ where v represents the instantaneous velocity vector and $\langle ... \rangle$ denotes the spatial average over the field of view.

Topological rearrangements were evaluated by identifying T1 neighbor-exchange events using the Tissue Analyzer plugin in `ImageJ/Fiji` with manual quality control to exclude false positives. A T1 transition was defined as the collapse of a cell–cell junction shared by two cells, followed by the formation of a new junction between two previously non-neighboring cells. The T1 transition rate was calculated as the number of exchange events per cell per hour. Cell segmentation and morphological analyses were performed using `Cellpose`. All quantitative image analyses were executed using `MATLAB` (version R2023a) or `ImageJ` (version 2.1.0/1.53c).

Glucose and Glutamine Tracing. MDCK cells were seeded at 30,000 cells/cm^2 in 6-well plates, ensuring that all samples for each time point were harvested simultaneously. For MPC inhibition, cells were treated with 2 μM UK5099 for 80 hours. Tracing was performed by culturing cells for 24 hours in either glucose-free DMEM (Gibco, 11966-025) or glutamine-free DMEM (Gibco, 11960-044), supplemented with [U-^{13}C]glucose (Cambridge Isotope Laboratories, CLM-1396-PK) or [U-^{13}C]glutamine, respectively.

Before metabolite extraction, medium was aspirated and cells were washed with cold 150 mM ammonium acetate (pH 7.3). Metabolites were extracted in 500 μL of cold 80% methanol, and cells were detached using a cell scraper. Norvaline (10 μL of 1 mM; Sigma) was added as an internal standard. Samples were vortexed briefly and centrifuged at 17,000 g for 5 minutes at 1 °C. The supernatant (400 μL) was transferred to glass vials (Fisher Scientific) and evaporated to dryness (EZ-2 Elite evaporator, Genevac). Dried extracts were resuspended in 300 μL of 50% acetonitrile:water, centrifuged for 15 minutes at 4 °C, and 5 μL of the clarified extract was injected onto a Luna NH_2 100 Å, 3 μm, 150 × 2.0 mm column (Phenomenex). Separation was performed on a Vanquish Flex UHPLC (Thermo Scientific) using mobile phases A (5 mM NH_4AcO, pH 9.9) and B (acetonitrile) at 200 μL/min. The gradient was 15%–95% A over 18 minutes, followed by 7 minutes at 95% A and re-equilibration to 15% A. Metabolites were analyzed on a Q Exactive mass spectrometer (Thermo Scientific) operated in polarity-switching full-scan mode (70–975 m/z, 70,000 resolution).

Metabolite quantification was performed in Maven (v8.1.27.11) using accurate mass ($<$5 ppm) and verified retention times. Values were normalized to cell number or protein content. Cell counts were determined from sacrificial wells using a Countess II automated cell counter and trypan blue exclusion (average of two replicates). Relative metabolite abundances were calculated as the sum of all isotopologue intensities, corrected for natural ^{13}C abundance using AccuCor. Data analysis was performed in `R` using custom scripts.

Seahorse Assay. Oxygen consumption rate (OCR) and extracellular acidification rate (ECAR) were measured using a Seahorse XFe24 Extracellular Flux Analyzer (Agilent Technologies). MDCK cells were seeded at 57,000 cells per well in Seahorse XF24 plates. On the day of the assay, cells were washed and incubated in unbuffered DMEM assay medium supplemented with 8 mM glucose, 2 mM glutamine, 1 mM sodium pyruvate, and 5 mM HEPES (pH 7.4). Mitochondrial stress tests were performed by sequential injection of 2 μM oligomycin (Port A), 0.75 μM FCCP (Port B), 1.35 μM FCCP (Port C), and a combination of 1 μM rotenone and 2 μM antimycin A (Port D). OCR and ECAR were recorded simultaneously throughout the assay. Rate measurements were normalized to cell number, determined by quantifying Hoechst-stained nuclei (10 μg/mL) using an Operetta High-Content Imaging System (PerkinElmer). ATP production rates were calculated and data was analyzed using previously established methods (5, 6).

Endocytosis and Macropinocytosis Assays. Cells were seeded in 4-chamber Ibidi µ-slides at a density of 30,000 cells/cm^2. To inhibit mitochondrial pyruvate uptake, cells were pre-treated with 5 μM UK5099 for three days. To inhibit macropinocytosis in jammed monolayers, cells were treated with 5 μM EIPA (MedChemExpress, HY-101840) for 2 h before analysis. Cells were then either fixed and immunostained for the early-endosome marker EEA1 or imaged by live-cell time-lapse microscopy for motility analysis. To examine the density dependence of EIPA-sensitive motility, EIPA was added at the time of seeding and maintained until analysis. Sparse cultures were analyzed 24 h after seeding, whereas crowded pre-jamming cultures were analyzed at approximately 95% confluence. At each density, cells were either fixed and immunostained for EEA1 or imaged by live-cell time-lapse microscopy. To inhibit dynamin-dependent endocytosis, cells were treated with 20 μM Dynasore for 12 h before analysis (7, 8).

For the macropinocytosis assay, we adapted the protocol established by Le et al. (9). Following EIPA treatment, the cells were put on ice and washed three times with cold PBS containing calcium and magnesium (Fisher Scientific, 14040-133). The cells were then incubated with 0.1 mg/mL FITC-Dextran 70 (Santa Cruz Biotechnology, sc-263323) in serum-free MEM-α medium for 15 minutes at 37 °C. To block further internalization, the cells were immediately put back on ice, washed three times with cold PBS+Ca/Mg to remove residual dextran. Cells were fixed with 4% paraformaldehyde (Thermo Scientific, 043368.9M) for 10 minutes at room temperature and stained with DAPI for 15 minutes while protected from light. Confocal imaging was performed on an NL5+ system mounted on a Zeiss AXIO Observer Z1 microscope using a 63× oil/1.40 NA objective. Fluorescence intensities for EEA1 and dextran-FITC were quantified with ImageJ/Fiji and normalized to the control condition.

Data Analysis and Statistics. All statistical analyses and data visualization were performed using GraphPad Prism (version 10) and MATLAB (version R2023a). Data are presented as mean ± standard deviation (SD). FDR values were estimated using Monte Carlo simulations by random permutation of X–Y data pairs. Statistical significance was defined as follows: $p > 0.05$, not significant (ns); $p \leq 0.05$, *; $p \leq 0.01$, **; $p \leq 0.001$, ***; and $p \leq 0.0001$, ****.

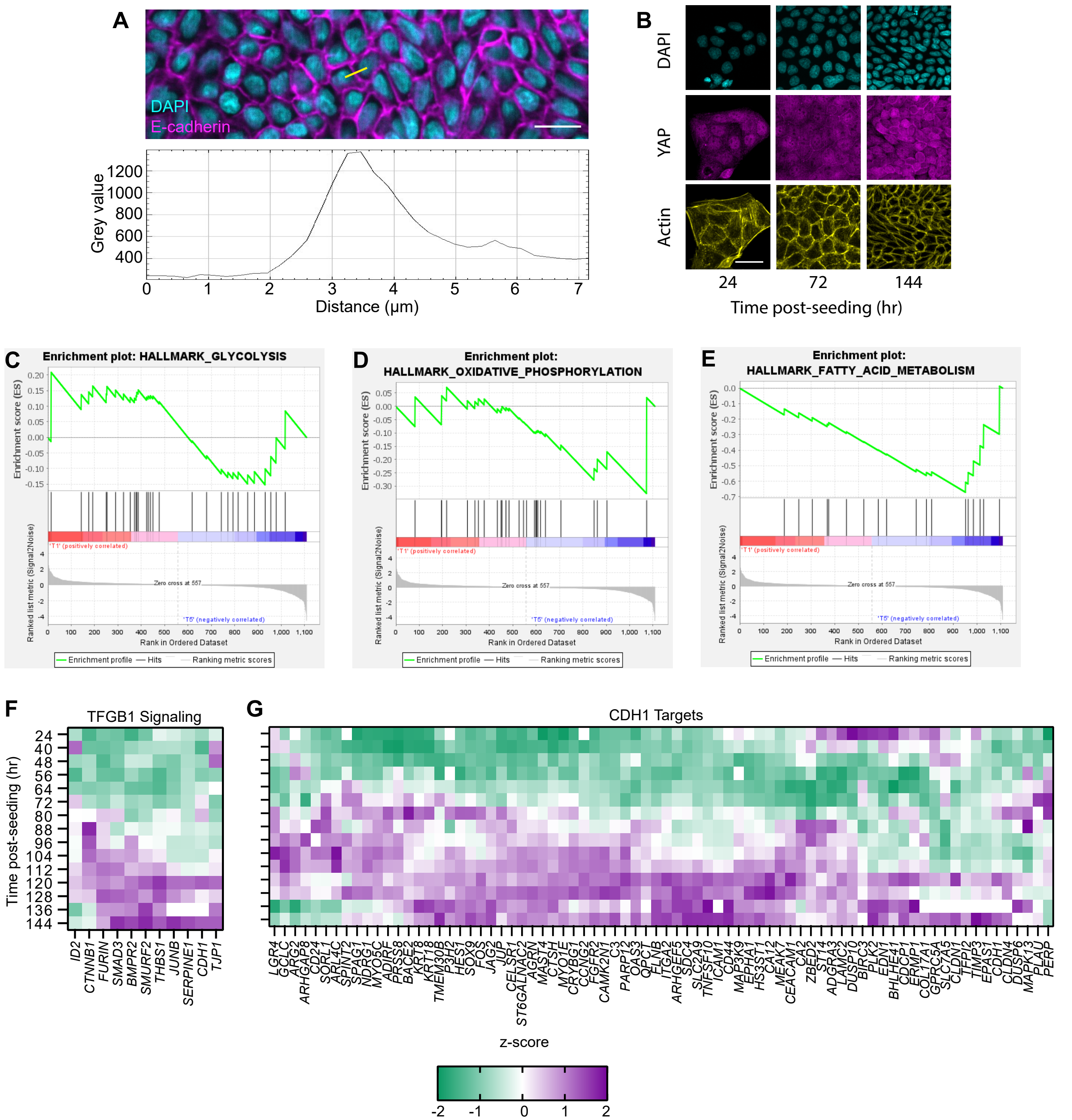

Appendix Figure 1. Characterization of epithelial junctions and metabolic transcriptomes. (A) Representative immunofluorescence image of E-cadherin in MDCK cells. The yellow line indicates the location of a cross-sectional 1D intensity profile (inset), used to calculate junctional contrast as the ratio of peak intensity to mean cytoplasmic background. Scale bar, 10 µm. (B) Single channel images of figure 1D. (C–E) GSEA enrichment plots between T1 and T5 datasets or metabolic hallmark gene sets: Glycolysis (C), Oxidative Phosphorylation (D), and Fatty Acid Metabolism (E). (F, G) Heatmaps displaying z-scored transcript levels for genes associated with TGF-β1 signaling (F) and CDH1-downstream targets (G)

.

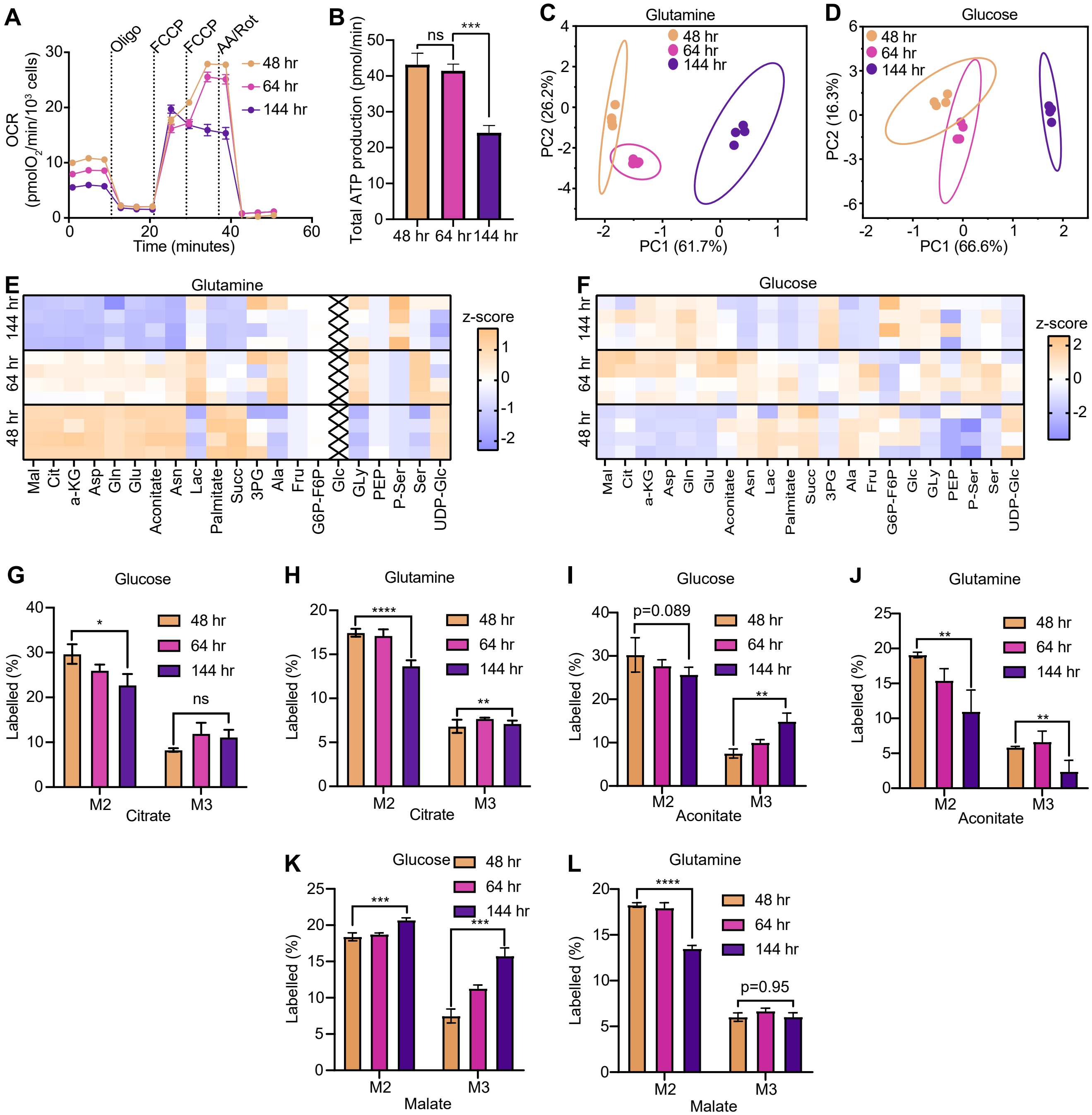

Appendix Figure 2. Metabolic remodeling during epithelial crowding. (A) Oxygen consumption rate (OCR) kinetic profiles during a mitochondrial stress test (Seahorse XF) across the indicated time points. OCR values are normalized to cell density. Vertical dashed lines indicate the sequential injection of oligomycin (Oligo), FCCP, and antimycin A/rotenone (AA/Rot). (B) Total ATP production 48, 64, and 144 hours after seeding. (C, D) Principal Component Analysis of fractional contributions from [U-13C] glutamine (C) and [U-13C] glucose (D) tracing. Distinct clustering by time point indicates temporal shifts in substrate utilization. (E, F) Heatmaps of z-scored fractional contributions for TCA cycle intermediates derived from glutamine (E) and glucose (F). (G–L) Mass isotopologue distributions (MIDs) for citrate (G, H), aconitate (I, J), and malate (K, L), highlighting M+2 and M+3 enrichments. Results are shown for glucose tracing (G, I, K) and glutamine tracing (H, J, L). ns, *, **, *** and **** correspond to p-values > 0.05, ≤ 0.05, ≤ 0.01, ≤ 0.001, and ≤ 0.0001, respectively. N = 4 independent biological replicates were used for all analysis. Each replicate represents a separate well.

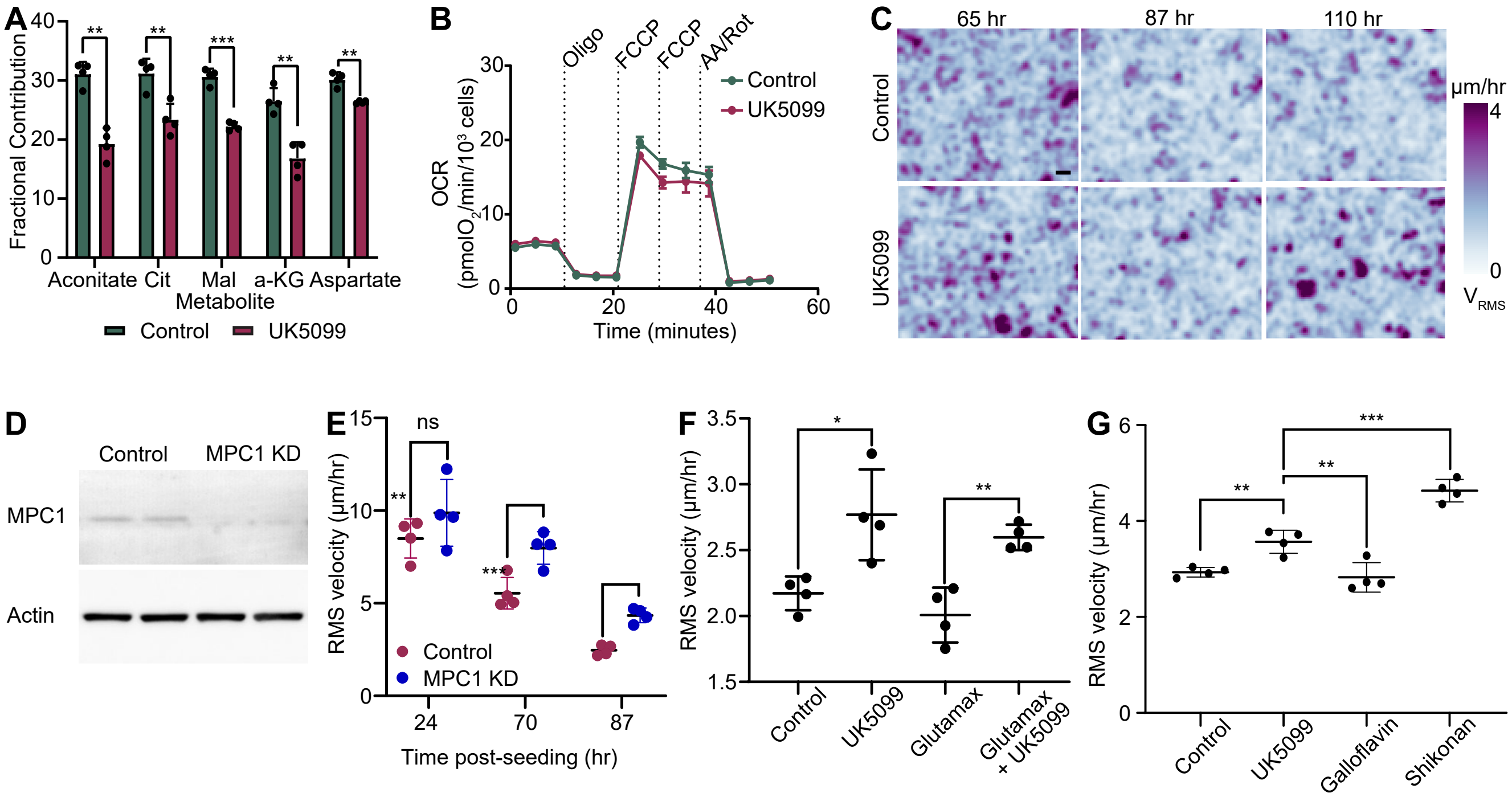

Appendix Figure 3. MPC inhibition via UK5099 promotes cell motility during epithelial crowding. (A) [U-13C] glucose tracing analysis of TCA cycle intermediates following treatment with the MPC inhibitor, UK5099. Reduced fractional contributions confirm successful inhibition of glucose-derived pyruvate entry into the TCA cycle. (B) Oxygen consumption rate (OCR) kinetic profiles (Seahorse XF) showing negligible impact of UK5099 on overall mitochondrial respiration. (C) Representative heatmaps of root-mean-square velocity (VRMS) at indicated time points for control and UK5099-treated monolayers. Scale bar, 10 μm. (D) Western blot validation of doxycycline-induced MPC1 knockdown (KD) in MDCK cells. Left, duplicate control samples (no doxycycline); right, duplicate MPC1 KD samples. Actin is shown as a loading control. (E) RMS velocity of control cells (no doxycycline) and MPC1 KD (doxycycline-induced) MDCK cells at three timepoints during jamming. (F) Comparison of motility phenotypes in media supplemented with L-glutamine versus GlutaMAX to examine potential glutamine degradation effects. (G) Perturbation of distinct branches of pyruvate metabolism differentiallyregulates epithelial cell velocity. Quantification of cell RMS velocity across control and metabolic inhibitor conditions (UK5099, Galloflavin, Shikonan). ns, *, **, *** and **** correspond to p-values > 0.05, ≤ 0.05, ≤ 0.01, ≤ 0.001, and ≤ 0.0001, respectively. N = 4 independent biological replicates were used for (A), (B), and (E-G), with each replicate representing a separate well. N = 2 independent biological replicates were used in (D).

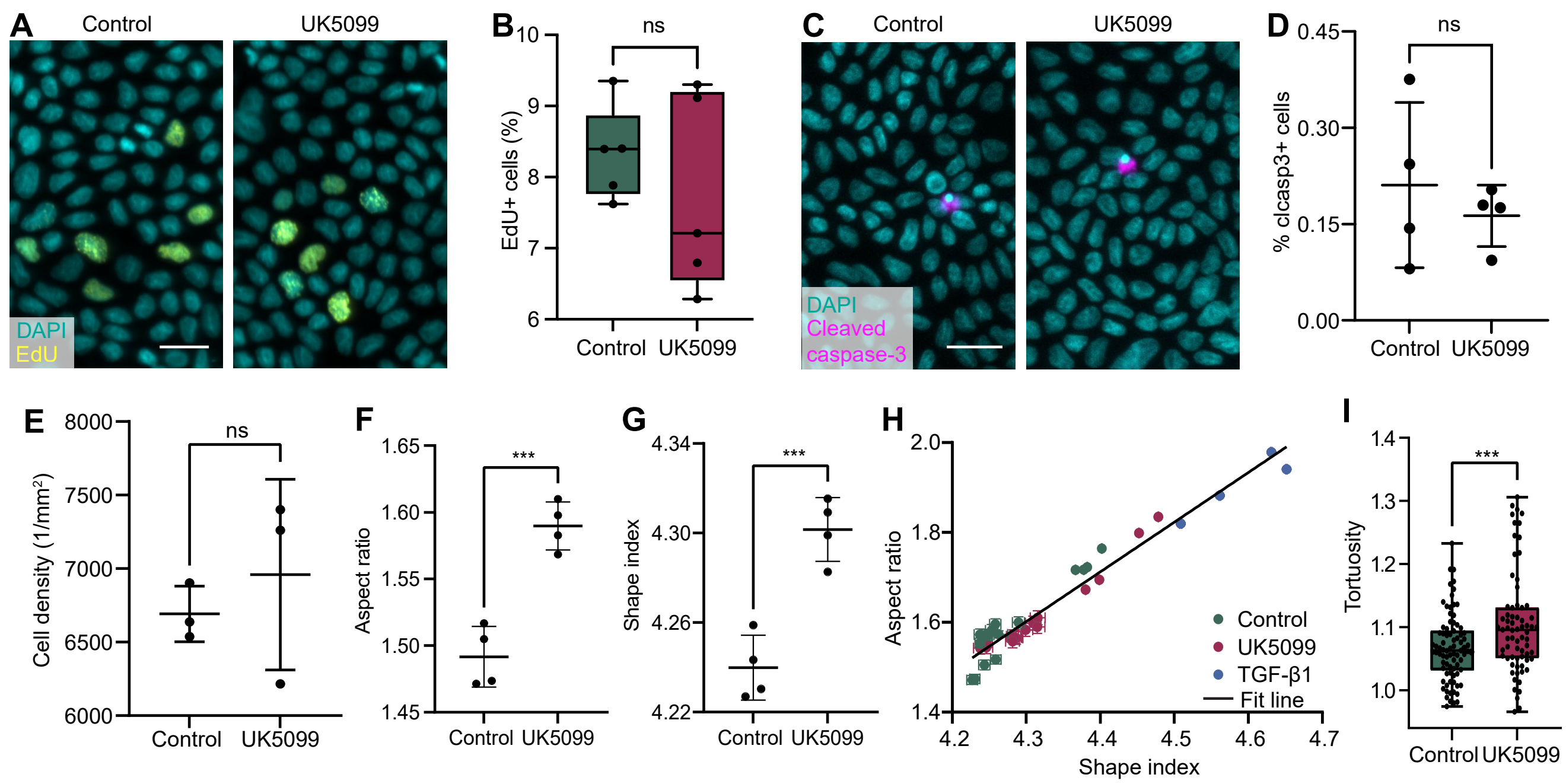

Appendix Figure 4. Impact of MPC inhibition on cell proliferation, apoptosis, and morphology. (A, B) Representative immunofluorescence images of EdU incorporation (A) and subsequent quantification (B), showing comparable proliferation rates between control and UK5099-treated cells. Measurements were conducted with at least n = 2000, where n is the number of cells analyzed. (C, D) Representative images of cleaved caspase-3 (clCASP3) staining (C) and quantification of clCASP3+ cells (D), indicating no significant induction of apoptosis upon UK5099 treatment. Measurements were conducted with at least n = 2000, where n is the number of cells analyzed. (E) Total cell density measurements across the indicated conditions. (F, G) Quantification of cell aspect ratio (F) and shape index (G) in control and UK5099 treated monolayers. Measurements were conducted with at least n = 500, where n is the number of cells analyzed. (H) Aspect ratio and shape index distributions for control, UK5099 treated, and TGF-β1 treated cells. (I) Analysis of E-cadherin junctional tortuosity. Tortuosity is defined as the ratio of the curvilinear junctional length to the Euclidean end-to-end distance. Measurements were conducted with n = 82 and 73 for control and UK5099, respectively, where n is the number of cells analyzed. ns, *, **, *** and **** correspond to p-values > 0.05, $\leq$ 0.05, $\leq$ 0.01, $\leq$ 0.001, and $\leq$ 0.0001, respectively. Scale bars, 50 µm. N = 5 independent biological replicates were used for B. N= 4 independent biological replicates were used for D-G. N = 36 indepent biological replicates were used for control and UK5099 treated cells in (H) while N = 4 were used for the TGF-β1 treated cells.

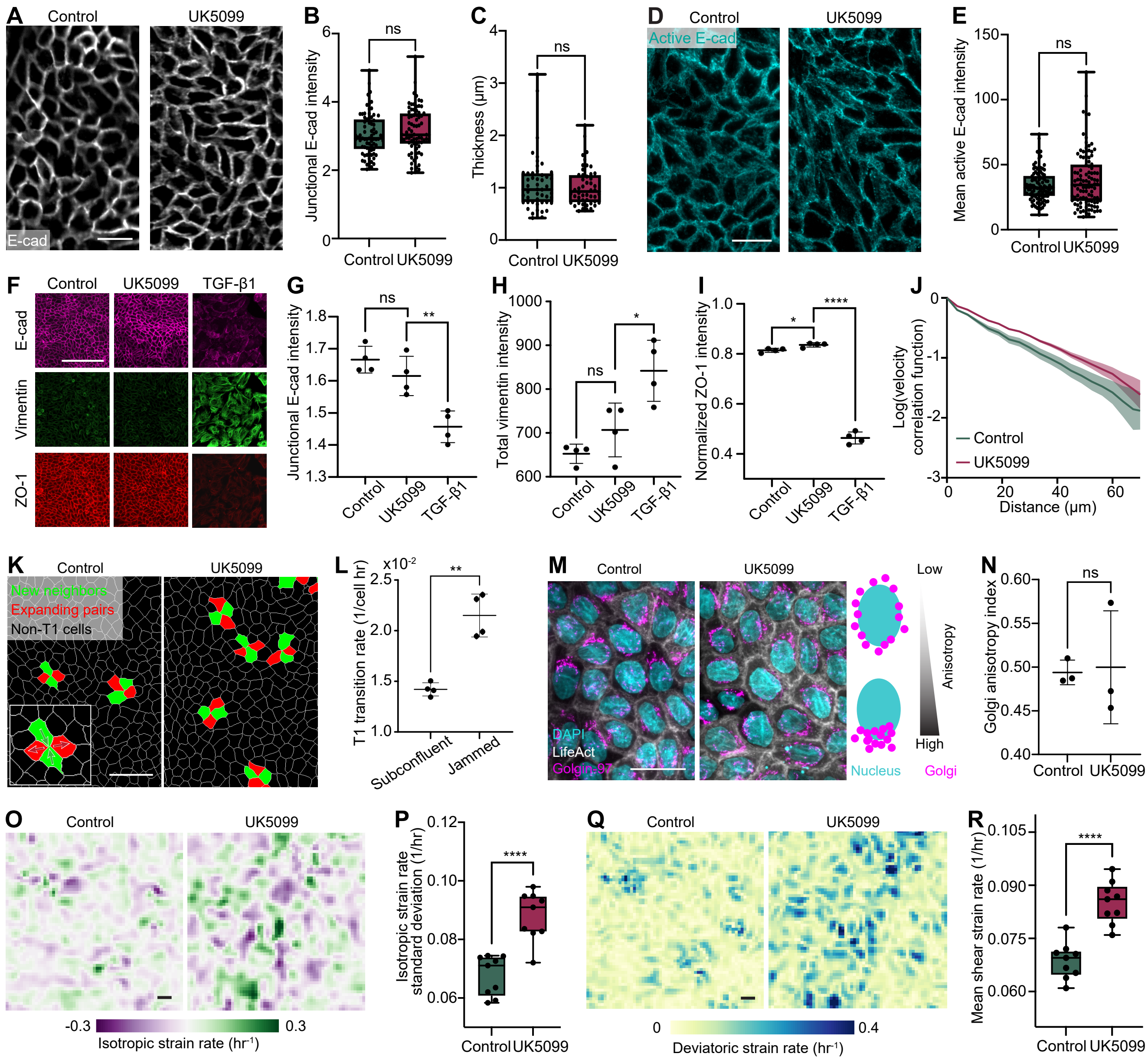

Appendix Figure 5. Morphological and kinematic characterization of MPC-inhibited monolayers. (A–E) Analysis of E-cadherin junctional morphology. Representative immunofluorescence images of E-cadherin (A) and quantification of junctional intensity (B) and junctional thickness (C). Measurements were conducted with n = 76 and 48 for B and C, respectively, where n is the number of cells analyzed. (D, E) Immunofluorescence of homophilically bound (active) E-cadherin (D) and corresponding quantification of mean fluorescence intensity (E). Measurements for (B-E) were conducted with n = 102, where n is the number of cells analyzed. (F) E-cad, vimentin, and ZO-1 single channel images for control, UK5099 treated, and TGF-β1 treated cells. (G-I) Quantifications of junctional E-cad intensity (G), total vimentin intensity (H), and normalized ZO-1 intensity (I) for images in (F). Measurements were conducted with n = 80, where n is the number of cells analyzed, for (G), and n= 4 for (H) and (I), where n is the number of FOVs analyzed. (J) Spatial velocity-velocity correlation functions as a function of intercellular distance. UK5099 treatment results in an increased correlation length, characterized by a slower spatial decay of the correlation metric. (K) Representative snapshot from TissueAnalyzer illustrating the identification and tracking of T1 transition events. (L) T1 transition rate in subconfluent and jammed cells. (M, N) Analysis of cell front-rear polarity via Golgin-97 immunofluorescence. (M) Representative images; the inset illustrates the method for calculating the intensity angular anisotropy index. (M) Quantification of the anisotropy index shows that MPC inhibition does not significantly alter global cell polarization. (O, P) Representative heatmaps of the isotropic strain rate derived from the velocity field (O) and corresponding quantification (P), showing enhanced volumetric expansion/contraction rates upon UK5099 treatment. (Q, R) Representative heatmaps of the deviatoric strain rate (Q) and quantification (R), indicating that MPC inhibition increases shear-like deformations within the monolayer. ns, *, **, *** and **** correspond to p-values > 0.05, ≤ 0.05, ≤ 0.01, ≤ 0.001, and ≤ 0.0001, respectively. Scale bar is 200 μm in (F) and 50 μm in (A), (D), (K), (M), (O), and (Q). N= 4 independent biological replicates were used for (G-I), and (L). N = 6 independent biological replicates were used for (J). N = 3 independent biological replicates were used for (N). N = 9 independent biological replicates were used for (P) and (R).

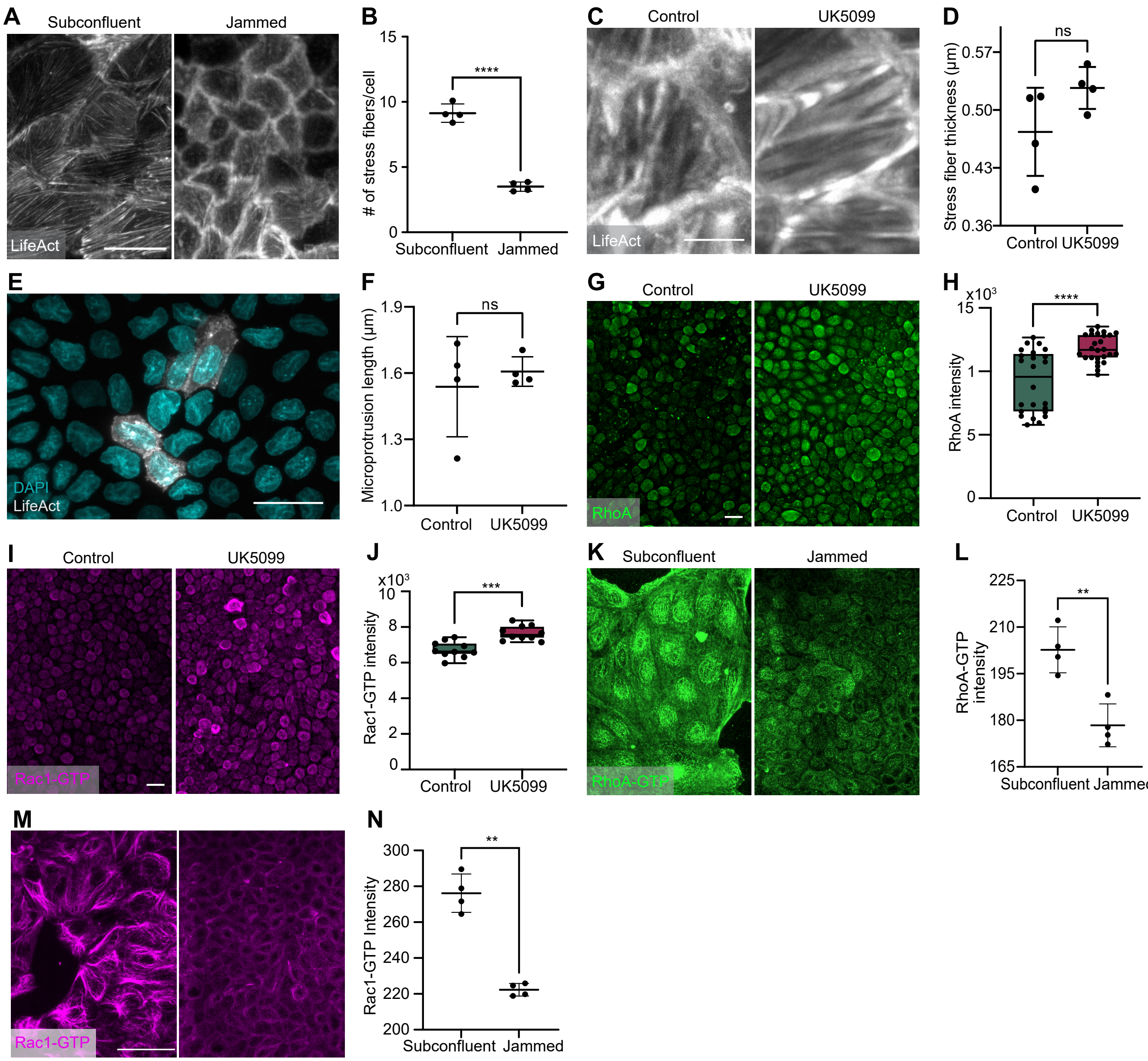

Appendix Figure 6. Cytoskeletal dynamics and Rho-family GTPase activity upon MPC inhibition. (A) Representative fluorescence images of LifeAct-GFP cells for stress fiber visualization when subconfluent and jammed. (B) Quantification of the number of stress fibers per cell for subconfluent and jammed cells. Measurements were conducted with n = 15, where n is the number of cells analyzed. (C, D) Representative fluorescence images of LifeAct-GFP (C) and quantification of stress fiber thickness (D), showing preserved contractile fiber morphology following UK5099 treatment. Measurements were conducted with n = 15, where n is the number of cells analyzed. (E) Representative field of view illustrating the cell density and distribution for sparse labeling experiments. (F) Quantification of microprotrusion length, indicating that MPC inhibition does not significantly alter protrusion length. Measurements were conducted with at least n = 25, where n is the number of cells analyzed. (G, H) Immunofluorescence images of total RhoA (G) and corresponding intensity quantification (H), showing an upregulation of RhoA expression in UK5099 treated cells. (I, J) Immunofluorescence images of Rac1-GTP (I) and corresponding quantification (J), demonstrating increased levels of the active GTP-bound form of Rac1 upon MPC inhibition. (K, L) Immunofluorescence images of RhoA-GTP (K) and corresponding intensity quantification (L) for subconfluent and jammed cells. (M, N) Immunofluorescence images of Rac1-GTP (M) and corresponding intensity quantification (N) for subconfluent (left) and jammed (right) cells. ns, *, **, *** and **** correspond to p-values > 0.05, ≤ 0.05, ≤ 0.01, ≤ 0.001, and ≤ 0.0001, respectively. Scale bar is 25 µm for (A) and 50 µm for (C), (E), (G), (I), (K), and (M). N = 4 independent biological replicates were analyzed in (B), (D), (F), (L), and (N). N = 10 independent biological replicates were analyzed in (J).

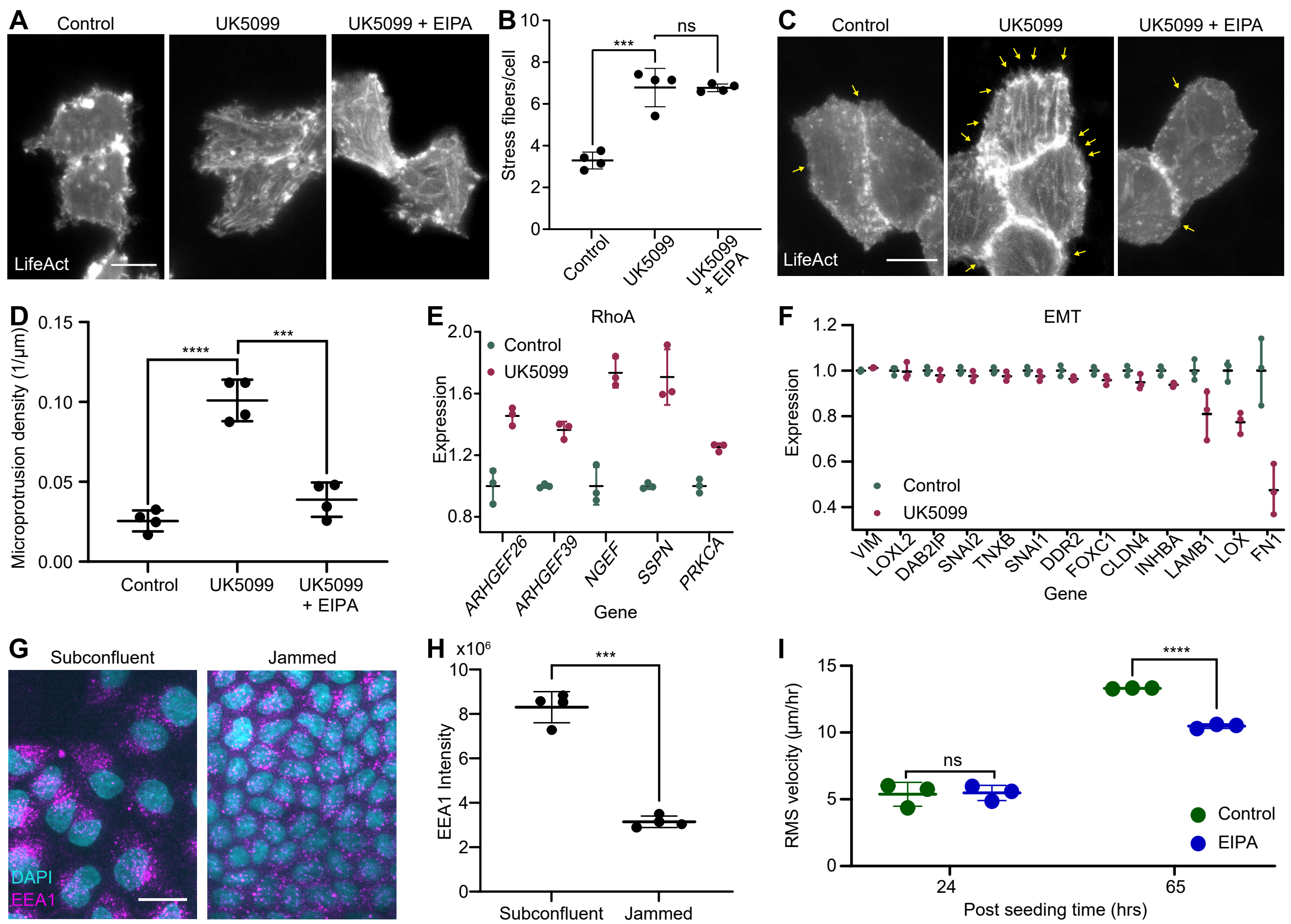

Appendix Figure 7. Impact of EIPA and MPC inhibition on cytoskeletal architecture and transcriptomic profiles. (A, B) Representative fluorescence images of LifeAct-GFP (A) and quantification of stress fiber density (B) in control, UK5099 treated, and UK5099 with EIPA treated monolayers. Na+/H+ exchanger exchange inhibition via EIPA does not significantly alter stress fiber formation. (C, D) Representative fluorescence images of LifeAct-GFP (C) and quantification of microprotrusion linear density (D). UK5099-induced microprotrusions (yellow arrows) are significantly suppressed following treatment with the Na+/H+ exchanger inhibitor EIPA. Measurements were performed with at least n = 3, where n is the number of cells analyzed. (E) Differential gene expression analysis via RNA-seq illustrating the upregulation of transcripts associated with RhoA signaling pathways. (F) Transcript levels of core EMT markers from RNA-seq data. No significant induction of a mesenchymal signature was observed, with specific markers (LAMB1, LOX, and FN1) showing modest downregulation upon UK5099 treatment. These results suggest that MPC inhibition-mediated motility occurs independently of a transition to a mesenchymal cell fate. (G) Representative EEA1 fluorescence images of subconfleunt and jammed cells. (H) Quantification of EEA1 intensity for subconfluent and jammed cells. (I) RMS velocity for EIPA treated cells during subconfluence. Scale bar for (G) is 20 µm. Scale bar is 10 µm for (A) and (C). ns, *, **, *** and **** correspond to p-values > 0.05, $\leq$ 0.05, $\leq$ 0.01, $\leq$ 0.001, and $\leq$ 0.0001, respectively. N = 4 independent biological replicates for (B), (D), (E), and (H). N = 3 independent biological replicates for (F) and (I).

Movie S1. Supplemental Video 1: Time-lapse phase-contrast microscopy showing MDCK cells undergoing jamming and the associated arrest of cell motility. The video corresponds to the root-mean-square (RMS) velocity quantification shown in Figure 1B.

Movie S2. TSupplemental Video 2: MPC inhibition enhances collective cell motility. Representative time-lapse phase-contrast microscopy (top) and corresponding root-mean-square (RMS) velocity fields (bottom) of cells treated with the MPC inhibitor UK5099. Arrow plots indicate the magnitude and direction of the velocity field, demonstrating increased motility compared to control conditions.

Movie S3. Supplemental Video 3: MPC inhibition enhances motility in RPTEC and RWPE1 cells. Representative time-lapse phase-contrast microscopy of RPTEC (top) and RWPE1 (bottom) cells treated with the MPC inhibitor UK5099. The videos correspond to the root-mean-square (RMS) velocity quantification in Figure 2M, demonstrating increased motility compared to control conditions.

Movie S4. Supplemental Video 4: Representative T1 transition in control cells. Time-lapse imaging capturing a neighbor exchange (T1 transition) within a control epithelial monolayer. Green contours highlight the "arriving" cell pair (gaining contact), while red contours highlight the "departing" cell pair (losing contact).

Movie S5. Supplemental Video 5: Representative T1 transition in UK5099-treated cells. Time-lapse imaging capturing a neighbor exchange (T1 transition) following MPC inhibition. Green contours highlight the arriving cell pair (gaining contact), and red contours highlight the departing cell pair (losing contact).

Movie S6. Supplemental Video 6: F-actin dynamics and baseline membrane ruffling in control cells. Time-lapse fluorescence microscopy of a sparsely labeled LifeAct-GFP cell, showing characteristic baseline membrane ruffling and cytoskeleton organization in the control group.

Movie S7. Supplemental Video 7: Enhanced membrane ruffling following UK5099 treatment. Time-lapse fluorescence microscopy of a sparsely labeled LifeAct-GFP cell, showing accelerated F-actin polymerization and enhanced membrane ruffling dynamics induced by MPC inhibition.

Movie S8. Supplemental Video 8: Inhibition of macropinocytosis or endocytosis reverses the increase in cell motility induced by MPC inhibition. Macropinocytosis was inhibited with EIPA, and endocytosis was inhibited with Dynasore. Representative time-lapse phase-contrast microscopy of control (top left), UK5099-treated (top right), UK5099 + EIPA-treated (bottom left), and UK5099 + Dynasore-treated (bottom right) cells. The videos correspond to the root-mean-square (RMS) velocity quantification shown in Figure 5J and demonstrate that inhibition of either macropinocytosis or endocytosis abolishes the UK5099-induced increase in cell motility.

References

1. M Martin, Cutadapt removes adapter sequences from high-throughput sequencing reads. *EMBnet. journal* **17**, 10–12 (2011).
2. A Dobin, Star manual 2.7. 0a. *Weill Cornell Medicine* **1252** (2019).
3. ME Ritchie, et al., limma powers differential expression analyses for rna-sequencing and microarray studies. *Nucleic acids research* **43**, e47–e47 (2015).
4. TT Onder, et al., Loss of e-cadherin promotes metastasis via multiple downstream transcriptional pathways. *Cancer research* **68**, 3645–3654 (2008).
5. BR Desousa, et al., Calculation of atp production rates using the seahorse xf analyzer. *EMBO reports* **24**, e56380 (2023).
6. AS Divakaruni, A Paradyse, DA Ferrick, AN Murphy, M Jastroch, Analysis and interpretation of microplate-based oxygen consumption and ph data in *Methods in enzymology.* (Elsevier) Vol. 547, pp. 309–354 (2014).
7. E Macia, et al., Dynasore, a cell-permeable inhibitor of dynamin. *Dev. cell* **10**, 839–850 (2006).
8. Q Ni, et al., Cell-nanoplastics association impacts cell proliferation and motility. *bioRxiv* (2026).
9. AH Le, LM Machesky, Image-based quantification of macropinocytosis using dextran uptake into cultured cells. *Bio-protocol* **12**, e4367–e4367 (2022).
